# Agent, Dose and Duration: The Unanswered Questions Behind Latency Antibiotic Therapy for Preterm Prelabour Rupture of Membranes

**DOI:** 10.3390/antibiotics15090926

**Published:** 2026-09-18

**Authors:** Iustina Petra Solomon Condriuc, Raluca Anamaria Mogoș, Gabriel Costachescu, Alexandru Cărăuleanu, Ioana Sadiye Scripcariu, Răzvan Socolov, Demetra Socolov

**Affiliations:** 1 Grigore T. Popa University of Medicine and Pharmacy, 700115 Iasi, Romania; 2 Department of Obstetrics and Gynecology, Cuza Voda Hospital, 700038 Iasi, Romania; 3 Department of Obstetrics and Gynecology, Clinical Hospital of Obstetrics and Gynecology “Elena Doamna”, 700038 Iasi, Romania

**Keywords:** preterm prelabour rupture of membranes, latency antibiotics, erythromycin, azithromycin, ampicillin, chorioamnionitis, expectant management, treatment duration

## Abstract

Latency antibiotic therapy is an established component of expectant management after preterm prelabour rupture of membranes (PPROMs). Pooled randomised evidence from 22 trials involving 6872 women and infants shows that antibiotics prolong pregnancy, reduce chorioamnionitis (risk ratio 0.66) and reduce neonatal infection (risk ratio 0.67), without a significant reduction in perinatal mortality. The evidence supporting the therapeutic principle is stronger than the evidence defining how the therapy should be configured. This narrative review examines the three decisions an obstetrician makes at the bedside, namely, which agent, at which dose and route, and for how long, and the comparative evidence behind each. Contemporary practice descends from two protocols: the ampicillin–erythromycin regimen of the NICHD Maternal–Fetal Medicine Units Network trial and the erythromycin arm of ORACLE I. Neither was designed to identify an optimal regimen. Substitution of azithromycin for erythromycin rests predominantly on observational cohorts together with one small open-label randomised comparison that pre-specified no non-inferiority margin and is therefore pragmatic and guideline-permitted rather than confirmed by an adequately powered randomised trial. No randomised dose comparison using clinical endpoints has been performed in PPROM, although randomised pharmacokinetic data exist for azithromycin. The seven-day course derives from a trial protocol rather than from a duration-ranging study, and the two randomised duration comparisons published since 2023 point in opposite directions. Guideline agreement reflects shared historical ancestry rather than independent confirmation of optimality. Adequately powered pragmatic trials, stratified by gestational age, remain the principal unmet need.

## 1. Introduction

Preterm prelabour rupture of membranes complicates approximately 2–3% of pregnancies and remains one of the principal identifiable antecedents of preterm birth [1]. Its contribution to the preterm birth burden varies with how preterm births are classified; in a contemporary Australian population cohort, PPROM accounted for 13.9% of preterm deliveries, with spontaneous preterm labour and iatrogenic delivery making up the remainder [2]. Globally, an estimated 13.4 million infants (95% credible interval 12.3–15.2 million) were born preterm in 2020, and rates have barely changed over a decade [3]. Against this background, any intervention that reliably prolongs a PPROM pregnancy carries substantial potential value.

Latency antibiotic therapy is such an intervention, and it is one of the few in obstetrics whose principle is supported by large placebo-controlled randomised trials. The Cochrane review of 22 trials involving 6872 women and infants found that antibiotics reduced chorioamnionitis (risk ratio [RR] 0.66, 95% confidence interval [CI] 0.46–0.96), birth within 48 h (RR 0.71, 95% CI 0.58–0.87) and birth within seven days (RR 0.79, 95% CI 0.71–0.89), as well as neonatal infection (RR 0.67, 95% CI 0.52–0.85), surfactant use, oxygen therapy and abnormal cerebral ultrasound findings, without a significant reduction in perinatal mortality [4]. Major obstetric societies recommend the intervention.

What the same body of evidence does not establish is how the intervention should be delivered. The obstetrician standing at the bedside of a woman who has ruptured membranes at 27 weeks must choose an antimicrobial agent or combination, a dose and route, and a treatment duration. These decisions are encountered routinely in obstetric practice worldwide, and each is currently made by convention.

This distinction is not merely academic. The populations, neonatal intensive care, viability thresholds and microbial ecology of 2026 differ from those of the late 1990s. A regimen that demonstrably outperformed placebo three decades ago may or may not be the regimen that best balances pregnancy prolongation, maternal safety, neonatal benefit and antimicrobial exposure today. The relevant question is not whether the historical trials were sound—they were—but whether the specific configuration of therapy they used has ever been subjected to comparative optimisation.

This narrative review is written from the perspective of the obstetrician and maternal–fetal medicine specialist managing expectant care after PPROM. It separates two questions that are frequently conflated. The first is relatively well answered: do latency antibiotics improve obstetric and neonatal outcomes in appropriately selected women? The second is not. Which agent, at which dose and route, and for what duration provides the optimal balance of benefit and harm? The review is organised around the three domains of the second question: agent, dose and route and duration. For each, we ask what randomised or comparative evidence exists, whether that evidence demonstrates superiority, formal non-inferiority or merely an absence of a statistically significant difference, and what an obstetrician can legitimately conclude from it.

Throughout, two further distinctions are maintained. Latency antibiotic therapy is prophylactic treatment of a woman without clinical infection and is categorically different from therapeutic antibiotics for established intra-amniotic infection, which is an indication for delivery rather than for continued expectant management [5]. It is also distinct from intrapartum group B streptococcal (GBS) prophylaxis, which has a different indication, a different target organism and a different timing [6].

Reviews of latency antibiotic therapy in PPROM already exist, and several are recent [7,8]. A systematic review of national and international guidelines has also catalogued the variation between societies [9]. This review differs in what it asks. Rather than summarising what is recommended, it separates the evidence for the therapeutic principle from the evidence for the therapeutic configuration. It then asks of each configuration decision what comparative evidence exists, and what that evidence licenses. The organising claim is that these two bodies of evidence differ substantially in evidential strength. That difference is obscured whenever a guideline’s operational detail is read as though it carried the authority of its headline recommendation.

## 2. Materials and Methods

### 2.1. Search Strategy

PubMed/MEDLINE, Embase, Scopus and the Cochrane Library were searched from database inception to 31 July 2026, using combinations of controlled vocabulary and free-text terms for preterm prelabour rupture of membranes, latency antibiotic therapy, the individual antimicrobial agents in current use, treatment duration and the maternal and neonatal outcomes relevant to this review. No language restriction was applied, and three non-English guideline documents were read in the original. Reference lists of key studies and of previous reviews were also screened. The search was intended to identify the landmark and comparative evidence bearing on the review question rather than to achieve systematic completeness.

ClinicalTrials.gov, the ISRCTN registry, the Clinical Trials Registry–India and the Thai Clinical Trials Registry were searched for completed and ongoing trials and were last accessed on 31 July 2026. The websites of ACOG, SMFM, RCOG, NICE, WHO, SOGC, RANZCOG, CNGOF, DGGG and FIGO were consulted for current recommendations and rechecked on 20 August 2026, the date to which the guideline positions in Table S2 refer.

### 2.2. Study Selection

Studies were selected purposively for their relevance to the clinical questions addressed here, with priority given to randomised trials, comparative observational studies, systematic reviews and current clinical guidelines. One author conducted the literature identification and selection. A second author independently verified the primary-source data and the bibliographic details underlying every comparative claim retained in the review. No independent duplicate screening of the complete search yield was undertaken. Retracted articles were excluded from the evidence base and are identified as retracted where the retraction bears on the argument. Conference abstracts were not used as primary sources for quantitative claims.

### 2.3. Appraisal and Presentation of Evidence

Consistent with the narrative design, no protocol was registered, no formal systematic eligibility criteria were applied, no risk-of-bias instrument was used and no quantitative synthesis was performed. The review makes no claim to systematic completeness. The fourth column of Table 1 classifies the comparative evidence available for each question by study design (direct randomised, indirect, observational, pharmacokinetic or none) rather than by a graded certainty rating. No GRADE assessment and no domain-by-domain risk-of-bias assessment were undertaken, and these descriptors must not be read as certainty ratings. Reporting was guided by the SANRA criteria for narrative review articles [10].

### 2.4. Scope

This review examines the configuration of latency antibiotic therapy. It does not re-examine whether latency antibiotics should be given at all; that question is addressed by the Cochrane review [4]. Diagnosis of membrane rupture, tocolysis, corticosteroid administration, magnesium sulphate and the timing of delivery are outside its scope, except where they bear on antibiotic choice.

## 3. From Trial Protocol to Standard Obstetric Practice

### 3.1. The Pre-1997 Evidence

Interest in antimicrobial therapy after PPROM grew from the observation that subclinical intrauterine infection precedes a substantial proportion of preterm membrane rupture. Between 1988 and 1999, at least a dozen small randomised trials tested this hypothesis, and they were strikingly heterogeneous in the agents used: ampicillin alone [41] and in GBS-colonised women [42], ampicillin combined with mezlocillin [43] or with erythromycin [44], penicillin [45,46], piperacillin [47], ampicillin–sulbactam [48], oral amoxicillin [49], co-amoxiclav [50] and erythromycin monotherapy [51,52]. Sample sizes were mostly in the low hundreds, outcome definitions differed, and few trials were powered for neonatal endpoints. Two meta-analyses pooled this material and supported a benefit [53,54], setting the stage for a definitive trial.

### 3.2. The Two Landmark Protocols

The NICHD Maternal–Fetal Medicine Units Network trial randomised 614 women with PPROM between 24 0/7 and 32 0/7 weeks to intravenous ampicillin 2 g every 6 h plus erythromycin 250 mg every 6 h for 48 h, followed by oral amoxicillin 250 mg every 8 h plus erythromycin base 333 mg every 8 h for five days, or to matching placebo [11]. The composite primary outcome occurred less often with antibiotics (44.1% versus 52.9%, *p* = 0.04), as did respiratory distress syndrome (40.5% versus 48.7%, *p* = 0.04) and necrotising enterocolitis (2.3% versus 5.8%, *p* = 0.03). In the GBS-negative cohort, the effect was more pronounced: the composite outcome occurred in 44.5% versus 54.5% (*p* = 0.03), overall sepsis in 8.4% versus 15.6% (*p* = 0.01) and pneumonia in 2.9% versus 7.0% (*p* = 0.04), with significant pregnancy prolongation. Two features of the protocol deserve emphasis because they are rarely carried forward when the regimen is quoted: antenatal corticosteroids had not been given before randomisation, and tocolysis and corticosteroids were prohibited afterwards. The trial therefore established the benefit of a specific seven-day regimen in a population managed without the co-interventions that are now routine.

ORACLE I randomised 4826 women with PPROM before 37 weeks in a 2 × 2 factorial design to erythromycin 250 mg four times daily, co-amoxiclav 325 mg four times daily, both or placebo, for up to ten days or until delivery [12]. The result is more qualified than it is usually reported to be. ORACLE I was a 2 × 2 factorial trial, so the comparisons below are marginal, pooled across the co-amoxiclav strata rather than simple two-arm contrasts. Among all 2415 infants in the erythromycin versus no-erythromycin comparison, the composite primary outcome of death, chronic lung disease or major cerebral abnormality occurred in 12.7% versus 15.2% (*p* = 0.08); the conventionally quoted reduction of 11.2% versus 14.4% (*p* = 0.02) applies to the 2260 singletons within that comparison. Co-amoxiclav did not confer benefit and was associated with necrotising enterocolitis, a signal later quantified in the Cochrane synthesis as RR 4.72 (95% CI 1.57–14.23) [4]. The companion trial in women in spontaneous preterm labour with intact membranes found no benefit from any regimen [55], and its seven-year follow-up reported more functional impairment (42.3% versus 38.3%; odds ratio [OR] 1.18, 95% CI 1.02–1.37) and more cerebral palsy after erythromycin (3.3% versus 1.7%; OR 1.93, 95% CI 1.21–3.09) and after co-amoxiclav (3.2% versus 1.9%; OR 1.69, 95% CI 1.07–2.67), with numbers needed to harm of 64 and 79, respectively [56]. The corresponding follow-up of the PPROM cohort found no such signal: functional impairment occurred in 38.3% versus 40.4% after erythromycin (OR 0.91, 95% CI 0.79–1.05) and in 40.6% versus 38.1% after co-amoxiclav (OR 1.11, 95% CI 0.96–1.28), with no effect on behaviour, medical conditions or educational attainment [57]. The distinction between the two ORACLE cohorts is frequently blurred in secondary literature; it should not be. The long-term safety signal belongs to intact membranes, and the reassurance belongs to PPROM.

### 3.3. How Two Protocols Became One Standard

The two trials were not comparative. They shared a control condition, not an intervention. One tested a β-lactam–macrolide combination given for seven days in a narrow gestational window; the other tested a macrolide, a β-lactam–inhibitor combination and their pairing, given for up to ten days across a much broader gestational range. Both demonstrated that antibiotics are better than nothing. Neither demonstrated that its own regimen was better than the other’s because neither was designed to do so.

What followed was adoption rather than optimisation. The NICHD regimen was incorporated essentially verbatim into North American guidance, and the ORACLE erythromycin arm into United Kingdom, WHO and much European guidance. Over the subsequent 25 years, no adequately powered randomised trial has directly compared these two strategies, and the most recent Cochrane update of the underlying question dates from 2013 [4]. A network meta-analysis of 20 randomised trials involving 7169 participants and 15 regimens found several regimens superior to placebo for chorioamnionitis—clindamycin plus gentamicin (RR 0.19, 95% CI 0.05–0.83), penicillin (RR 0.31, 95% CI 0.16–0.60), ampicillin (RR 0.52, 95% CI 0.34–0.81) and erythromycin plus amoxicillin (RR 0.71, 95% CI 0.55–0.92)—but concluded that none was clearly and consistently superior, with evidence quality moderate to very low [14]. That conclusion, drawn from the totality of the randomised literature, is the most defensible summary of where regimen selection currently stands.

**Figure 1:**
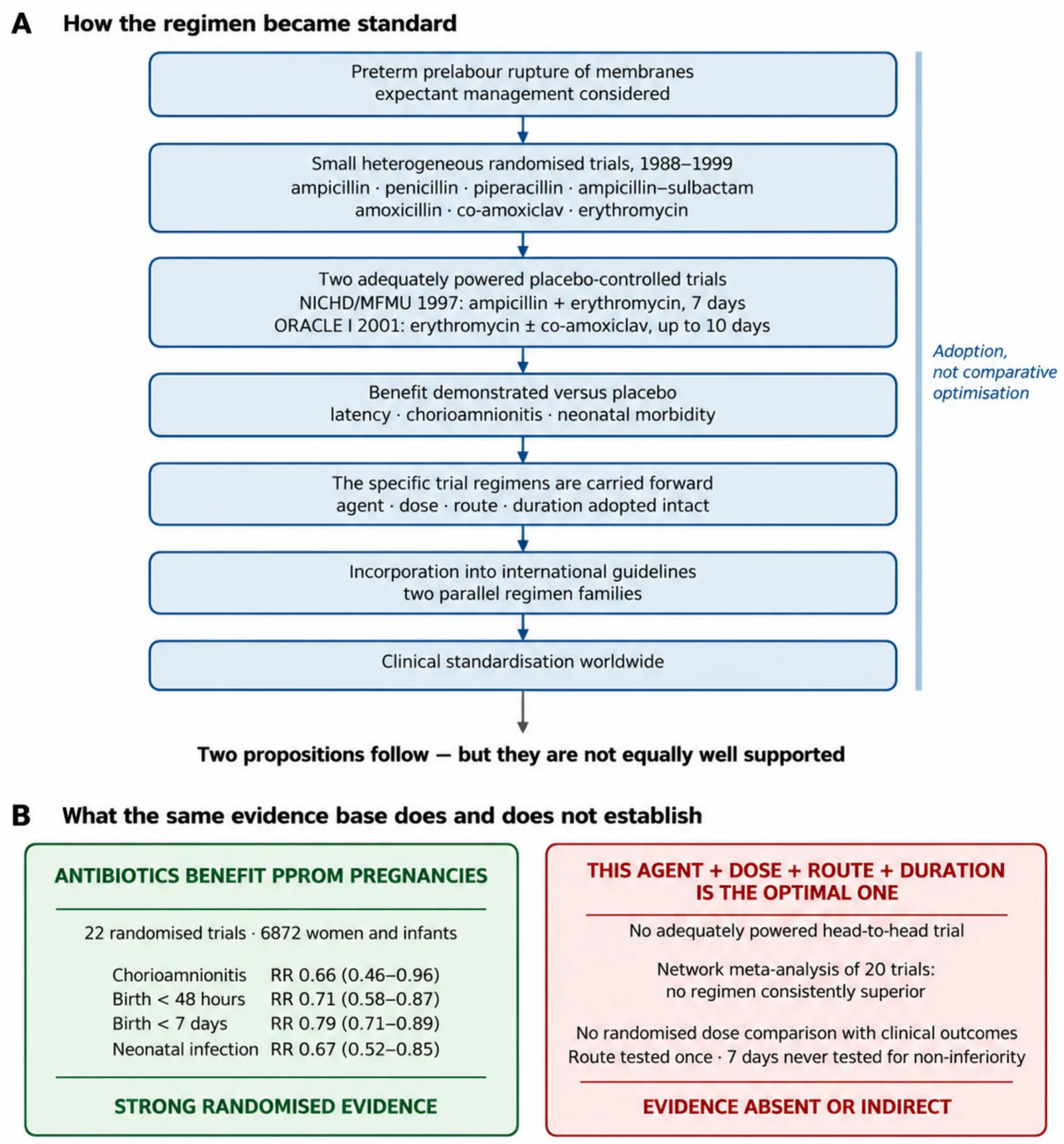
From evidence to convention: how a successful latency antibiotic protocol became standard obstetric practice. (**A**) The sequence by which contemporary practice was established. Preterm prelabour rupture of membranes (PPROM) prompted a series of small, heterogeneous randomised trials of diverse antimicrobial agents between 1988 and 1999 [41,42,43,44,45,46,47,48,49,50,51,52], summarised in two meta-analyses [53,54]. Two adequately powered placebo-controlled trials followed: the NICHD Maternal–Fetal Medicine Units Network trial of a seven-day ampicillin–erythromycin regimen [11] and ORACLE I, which tested erythromycin, co-amoxiclav or both for up to ten days [12]. Each demonstrated benefit against placebo, and each trial’s specific regimen was then adopted into guidelines—the NICHD combination in North America, the ORACLE erythromycin arm in the United Kingdom, WHO and much of Europe—and thereafter into routine practice. (**B**) Two propositions that this pathway conflates. The proposition that antibiotics improve outcomes in PPROM is supported by 22 randomised trials involving 6872 women and infants, with consistent reductions in chorioamnionitis, birth within 48 h and seven days, and neonatal infection [4]. The proposition that this particular combination of agent, dose, route and duration is optimal is supported by no adequately powered head-to-head comparison; a network meta-analysis of 20 randomised trials found no regimen consistently superior [14]. NICHD, Eunice Kennedy Shriver National Institute of Child Health and Human Development.

The consequence is visible in practice. A survey of 235 clinicians in Australia and Aotearoa New Zealand found that 97% routinely prescribed antibiotics after PPROM, yet documented 90 different regimens; erythromycin was used by 88% and a penicillin by 46% [40]. A Korean survey of 113 obstetricians found routine cephalosporin use by 81.4% [58]. Uniform endorsement of a principle has not produced uniform practice, because the guidelines are not uniform about the specifics, and the specifics have not been tested against each other. Figure 1 traces that path from trial to convention. Detailed characteristics of the landmark and contemporary studies on which the three domains examined below depend are provided in Table S1.

## 4. Agent: Choosing the Antimicrobial Strategy

### 4.1. Two Dominant Regimen Lineages, One Evidence Base

Most contemporary guideline strategies descend from two dominant historical lineages: a β-lactam–macrolide combination given for seven days, derived from the NICHD trial, and macrolide monotherapy for up to ten days or until labour, derived from ORACLE I. These are not the only options in use. Several societies also accept β-lactam monotherapy, third-generation cephalosporin monotherapy or hybrid schedules (Table S2). An obstetrician moving between a North American and a British unit will encounter both dominant lineages, prescribed with equal confidence, on the strength of the same two trials (Section 11).

Direct comparison between these two lineages is limited to observational data. The largest identified is a retrospective multicentre Canadian cohort of 669 PPROM pregnancies between 18 and 34 weeks. Macrolide monotherapy was associated with shorter latency than a β-lactam–macrolide combination (adjusted OR for latency ≤ 7 days 1.9, 95% CI 1.3–2.8; adjusted mean latency −6.7 days, 95% CI −9.3 to −4.1). Clinical chorioamnionitis was more frequent in the macrolide-alone group (37% versus 14–25% across the comparator regimens, *p* = 0.001), as were histological chorioamnionitis (74% versus 52–61%, *p* < 0.001) and neonatal intraventricular haemorrhage (23% versus 11–13%, *p* = 0.009) [13]. The finding is directionally consistent with the network meta-analysis [14], but it is confounded by indication: macrolide monotherapy was used predominantly in women with reported penicillin allergy, a group that differs systematically from the remainder. Because treatment was selected by allergy status rather than by randomisation, and reported allergy is itself associated with the obstetric and neonatal outcomes being compared, this design cannot separate the effect of the regimen from the characteristics of the women who received it and cannot establish a causal advantage of combination therapy. The result should therefore be read as a strong argument for formal comparison, not as proof of superiority. Randomised comparisons between named regimens, as distinct from comparisons between the two dominant lineages, remain rare and small. The most recent, published in 2026, randomised 40 women with PPROM at 34–37 weeks to ampicillin–sulbactam with azithromycin and amoxicillin, or to ceftriaxone with clarithromycin and amoxicillin. Neither neonatal intensive care stay (8.3 ± 6.7 versus 4.3 ± 1.9 days, *p* = 0.356) nor clinical chorioamnionitis (20.0% versus 5.0%, *p* = 0.141) differed significantly [59]. The trial enrolled 40 women, reports no sample-size calculation and was registered after recruitment began (NCT07148622). Its population lies in a window in which several societies do not recommend latency antibiotics, so its external validity for expectant management before 34 weeks is limited. Differences of the magnitude reported are precisely what a trial of 40 participants cannot resolve.

### 4.2. Erythromycin, Azithromycin and the Limits of Substitution

Erythromycin has been progressively replaced by azithromycin in many units, driven by supply interruption, gastrointestinal intolerance, dosing convenience and cost; a modelled analysis of 981 PPROM pregnancies estimated a reduction of more than 95% in drug acquisition cost with either azithromycin regimen [60]. The pharmacological rationale is reasonable. After a single oral 1 g dose, azithromycin concentrations in myometrium, adipose tissue and placenta exceed 500 ng/mL and persist to 72 h, whereas maternal serum concentrations peak at 311 ng/mL at 6 h and fall to 63 ng/mL by 24 h; amniotic fluid concentrations peak at 151 ng/mL and then decline [61]. The rationale is tissue distribution and dosing convenience rather than superior fetal exposure: transplacental transfer is limited for all three macrolides studied in pregnancy and did not differ significantly between erythromycin, roxithromycin and azithromycin (3.0%, 4.3% and 2.6%, respectively) [62]. Azithromycin’s pharmacokinetics in pregnancy have been reviewed in detail [63].

Clinical evidence is predominantly observational. Three cohorts detected no latency difference: 9.4 ± 10.0 versus 9.6 ± 13.2 days in 168 pregnancies, with power for only a five-day difference (*p* = 0.40) [64]; median 5.86 (IQR 3.12–12.05) versus 6.37 (IQR 3.59–10.93) days in 162 pregnancies (*p* = 0.75) [65]; and median 3.70 versus 3.23 days in 720 women, of whom only 56 received azithromycin (*p* = 0.287) [16]. Further comparative cohorts have reported similar findings [66]. A prospective cohort of 310 pregnancies found unchanged latency but lower clinical chorioamnionitis (13.4% versus 25%, *p* = 0.010; adjusted RR 0.51, 95% CI 0.30–0.89), postpartum endometritis (adjusted RR 0.46, 95% CI 0.27–0.76) and neonatal sepsis (adjusted RR 0.32, 95% CI 0.14–0.76) with azithromycin [67]. One matched Irish cohort found longer latency with amoxicillin plus azithromycin (median 5.5 versus 2 days, *p* < 0.001), a result at odds with the others and best regarded as an outlier [68]. Pooling five cohorts (1289 women) gave a latency mean difference of 0.07 days (95% CI −0.45 to 0.60) and a chorioamnionitis OR of 0.53 (95% CI 0.39–0.71) favouring azithromycin [15].

One randomised comparison of the two macrolides has been published. Musavi et al. allocated 194 women with ruptured membranes to oral azithromycin 1 g or to erythromycin, each combined with ampicillin, in an open-label trial at a single Iranian centre [17]. The trial enrolled a mixed preterm and near-term population, pre-specified no non-inferiority margin and was not indexed in MEDLINE. Several further comparisons are registered, and at least one has completed recruitment (NCT01556334, NCT05328817, NCT06273891, NCT07394725). The substitution has therefore been randomised, if only once and inadequately. What is missing is a trial large enough, and designed tightly enough, to exclude a clinically important difference.

Two points follow. A mean latency difference of 0.07 days, with a confidence interval spanning less than half a day either way, is a tight null; it is nonetheless pooled from non-randomised studies with no pre-specified non-inferiority margin, and it does not license the word “equivalent”. The chorioamnionitis signal is biologically plausible but vulnerable to the pre/post design of most contributing studies, in which azithromycin cohorts are systematically more recent. The direction of the observational evidence is also less uniform than the pooled estimate suggests. In the Thai cohort of 720 women, postpartum endometritis (3.6% versus 0.2%, *p* = 0.017) and intraventricular haemorrhage (3.6% versus 0%, *p* = 0.006) were both more frequent after azithromycin [16]. Both rest on single-figure event counts and neither analysis was adjusted. They are the principal countervailing signals identified in this review and warrant surveillance in any trial that is eventually mounted. A phase II randomised trial comparing ampicillin–sulbactam plus azithromycin with ampicillin–sulbactam plus erythromycin in PPROM at 22–27 weeks is under way; its primary outcome is a composite of moderate or severe bronchopulmonary dysplasia at 36 weeks’ postmenstrual age, intrauterine fetal death or infant death [18]. Until it and comparable trials report, azithromycin substitution is a reasonable pragmatic choice supported predominantly by observational evidence and by guideline permission [1,34], not a demonstrated improvement.

### 4.3. The β-Lactam Component and What the NEC Signal Actually Means

The necrotising enterocolitis signal associated with co-amoxiclav is specific and statistically significant (RR 4.72, 95% CI 1.57–14.23) [4,12], and guidelines consistently advise against it. It is sometimes generalised into a broader suspicion of β-lactams in PPROM. That generalisation is not supported: in the NICHD trial the ampicillin-containing regimen was associated with *less* necrotising enterocolitis than placebo (2.3% versus 5.8%, *p* = 0.03) [11]. The signal belongs to clavulanate-containing therapy, not to β-lactams as a class.

Whether a broader-spectrum β-lactam performs better than ampicillin is a legitimate and largely untested question. A small randomised trial (*n* = 84) comparing cefuroxime plus roxithromycin with ampicillin plus roxithromycin reported a longer median latency with the cephalosporin regimen (4.63 versus 2.30 days, *p* = 0.039) [69]. The same group then conducted a three-centre randomised trial in 124 women with rupture before 37 weeks, comparing cefuroxime plus roxithromycin with ampicillin plus roxithromycin [70]. Maternal infectious morbidity was lower in the cefuroxime arm (6.5% versus 17.7%, one-sided *p* = 0.048), and the organisms recovered from placental, membrane, cord and uterine cultures differed between arms. A one-sided test at the conventional threshold is a weak basis for claiming superiority, and the trial was not powered for neonatal outcomes. It is nonetheless the only multicentre randomised comparison of β-lactam backbones that we identified. A subsequent cohort from the same group, comparing cefuroxime plus azithromycin with ampicillin plus roxithromycin in 170 pregnancies, found less clinical chorioamnionitis (1.1% versus 8.4%, *p* = 0.046), fewer maternal postpartum infections and fewer ampicillin-resistant *Enterobacteriaceae* (10% versus 23%, *p* = 0.027) with the newer protocol [71]. The cohort is a single-group and sequential-era and cannot establish superiority, but together with the two trials they identify a plausible direction of enquiry that is reinforced by resistance data discussed below.

A more aggressive strategy has been evaluated in a two-era Korean cohort of 314 pregnancies, in which ceftriaxone, clarithromycin and metronidazole were continued until delivery. Median latency was 23 days (IQR 10–51) against 12 days (IQR 5–52) in the earlier era, with an adjusted hazard ratio for delivery of 0.69 (95% CI 0.49–0.96) and delivery within seven days in 20.2% versus 43.6% (adjusted OR 0.40, 95% CI 0.21–0.77). Chorioamnionitis, funisitis and intraventricular haemorrhage were all less frequent, and the differences were concentrated in pregnancies with documented intra-amniotic infection or inflammation [72]. The same group reported eradication of intra-amniotic inflammation or infection in 33.3% of women treated with the newer regimen versus none with the older one [73]. The comparison spans 1993–2003 and 2003–2012, an interval over which neonatal intensive care changed substantially; these findings justify a trial rather than a change in practice.

### 4.4. Anaerobic Coverage and Spectrum

We identified no randomised trial testing the addition of dedicated anaerobic coverage to a standard latency regimen in PPROM. Metronidazole and clindamycin appear only within composite regimens rather than as tested additions to a standard backbone, and French national guidance advises against both, along with co-amoxiclav, aminoglycosides, glycopeptides and first- and second-generation cephalosporins [31]. The underlying microbiology offers only partial direction. Microorganism-associated intra-amniotic inflammation occurs in approximately 21% of PPROM pregnancies and sterile inflammation in a further 4% [74], and antibiotics do not reliably eradicate established intra-amniotic infection: in one series with paired amniocentesis, six of seven women with initially positive cultures remained culture-positive, and 32% of initially culture-negative women developed new intra-amniotic inflammation despite treatment [75]. A broader spectrum is therefore not automatically better spectrum. Which spectrum is appropriate is therefore an empirical question and one that comparative studies can answer only if they measure that microbiological as well as clinical outcomes (Section 13). How much of that microbiology is visible, however, depends on how it is sought. *Ureaplasma* and *Mycoplasma* species are fastidious, require specialised media and are poorly recovered by routine culture, so a negative conventional culture does not establish that the amniotic cavity is free of microorganisms [76]. Molecular assays detect microbial nucleic acid in amniotic fluid that culture reports as negative and identify fastidious organisms that culture-based surveys miss altogether [77]. Prevalence estimates, and apparent eradication rates after treatment, are therefore partly a function of the detection method, and culture-based and molecular studies should not be treated as interchangeable without regard to the analytical sensitivity of each and what each actually measures. Detection of nucleic acid does not by itself establish viable organisms or clinically significant intra-amniotic infection; the two approaches are complementary, and neither is a gold standard on its own.

### 4.5. Ureaplasma, Mycoplasma and the Rationale for the Macrolide

Where a macrolide is included in a latency regimen, it is directed, whether or not the guideline says so, at organisms a β-lactam cannot reach. *Ureaplasma* species and *Mycoplasma hominis* have no cell wall and are intrinsically resistant to β-lactams. They are also the organisms most often recovered from the amniotic cavity after membrane rupture. Among women with PPROM undergoing amniocentesis, *Ureaplasma* species dominate the amniotic fluid isolates, and microbial loads are higher when intra-amniotic inflammation is present than when the cavity is colonised without inflammation [74]. The same organisms predominate outside the cavity: in 293 PPROM pregnancies, *Ureaplasma* species were the commonest pathogen both in vaginal swabs at diagnosis and in placental swabs when chorioamnionitis followed [78].

Three consequences follow for regimen selection.

The first concerns which macrolide. Transplacental transfer is limited and similar across erythromycin, roxithromycin and azithromycin [62]. Clarithromycin achieves greater placental passage than other macrolides in an ex vivo perfusion model, with a mean transplacental transfer of 6.1% [79]. Whether any macrolide attains inhibitory concentrations for *Ureaplasma* within the foetal compartment has not been established in vivo. For an ascending infection, however, the pharmacodynamic targets are maternal tissue and amniotic fluid rather than foetal serum. The dosing question examined in Section 5 therefore bears more directly on efficacy than transplacental transfer does. It also explains why the 500 ng/mL threshold for *Ureaplasma* is the demanding one in the simulation work discussed there [25], although that concentration is a susceptibility benchmark used in simulation rather than a validated pharmacodynamic target for latency therapy.

The second concerns eradication. Maternal azithromycin clears *Ureaplasma parvum* from amniotic fluid in non-human primate models of intra-amniotic infection [80]. Clinical results are less complete. *Ureaplasma* has been recovered from placental swabs despite azithromycin having been started at diagnosis [78], vaginal *Ureaplasma* persists in a proportion of treated women [81] and macrolide resistance among genital mycoplasmas is increasingly reported. The organisms grouped under that label do not, however, share a susceptibility phenotype. *Mycoplasma hominis* is intrinsically resistant to the 14- and 15-membered macrolides used in PPROM regimens, erythromycin and azithromycin among them [82], whereas *Ureaplasma* species are generally macrolide-susceptible and acquired resistance in them is variable rather than uniform; reported resistance prevalence differs markedly between countries and between study populations, and part of that spread reflects differences in susceptibility testing method and breakpoint definition rather than true biological variation [83]. The implication is cautious rather than prescriptive. The macrolide component of the historical regimens was selected in a different resistance landscape, so how much of the intended non-β-lactam coverage a given regimen delivers may depend on local species distribution and susceptibility patterns; that is an argument for contemporary microbiological characterisation alongside clinical comparison, not for abandoning macrolide-based latency therapy, whose benefit against placebo rests on randomised evidence [4,12]. Prophylaxis reduces the rate of intra-amniotic inflammation in PPROM without abolishing it [84], which is consistent with the older observation that established intra-amniotic infection is rarely eradicated [75]. Apparent eradication in these studies is itself method-dependent, since culture and molecular detection do not report the same thing (Section 4.4).

The third concerns the endpoint. Pulmonary *Ureaplasma* colonisation is associated with bronchopulmonary dysplasia, and neonatal azithromycin has been studied for that indication [85]. This is the reasoning behind the primary outcome of the ongoing Japanese phase II trial, which is moderate or severe bronchopulmonary dysplasia at 36 weeks’ postmenstrual age rather than an infectious endpoint [18]. Latency and clinical chorioamnionitis, which dominate the observational comparisons of macrolide regimens, may be poorly sensitive to the organism the macrolide is there to cover.

None of this identifies a preferred macrolide, but it explains why a comparison between macrolides that measures only latency is unlikely to detect the difference that would justify the choice.

## 5. Dose and Route: Protocol Inheritance Rather than Evidence-Based Optimisation

The doses in current use can be traced to two documents. The NICHD protocol specified ampicillin 2 g intravenously every 6 h with erythromycin 250 mg every 6 h for 48 h, followed by amoxicillin 250 mg every 8 h with erythromycin base 333 mg every 8 h for five days [11]. ORACLE I specified erythromycin 250 mg four times daily orally [12]. Both sets of figures appear in current guidance essentially unchanged [1,86,87,88]. Among the trials identified, none has compared two doses of ampicillin, two doses of amoxicillin or two doses of erythromycin. The doses are protocol-derived rather than evidence-optimised; they were not selected through dose-ranging comparison. Pregnancy itself supplies a mechanistic reason to ask whether doses inherited from these protocols achieve contemporary pharmacokinetic/pharmacodynamic (PK/PD) targets. Glomerular filtration rate and renal clearance rise, total body water and plasma volume expand so that the apparent volume of distribution increases, and plasma protein binding falls, raising the unbound fraction; these changes are documented across drug classes, including antibacterial agents, and several of them vary in magnitude with gestational age [21]. Their net effect on any individual agent is not uniform and cannot be assumed from first principles. That is precisely the point. The NICHD and ORACLE doses have not been shown to be inadequate, and nothing here implies that they are; but neither have they been evaluated against a defined target-attainment criterion in a contemporary pregnant population. Pregnancy-altered pharmacokinetics therefore provide a mechanistic rationale for formal reassessment of target attainment, not a basis for altering prescribed doses.

The single exception concerns azithromycin, where the substitution debate created a genuine dosing question: a single 1 g oral dose or a multi-day course? The ADAPT (Azithromycin Dosing and PPROM Treatment) randomised phase I trial compared 1 g once with 500 mg daily for seven days [24]. Beyond 96 h, median amniotic fluid concentrations were higher with daily dosing. The daily-dose figure was 46 ng/mL (interquartile range 23–196) compared with 11 ng/mL (interquartile range 7–56) after the single dose, a median difference of −27 ng/mL (interquartile range −154 to −1, *p* = 0.03). Plasma trough concentrations remained elevated on daily dosing while falling to near-undetectable levels after the single dose. Recruitment stopped after six women, and no clinical outcomes were reported.

That cohort (*n* = 6) has since been extended into a population pharmacokinetic analysis with simulation of alternative oral schedules [25]. The benchmarks are the minimum inhibitory concentrations for the organisms a macrolide is prescribed to cover. These are approximately 20 ng/mL for *Streptococcus agalactiae*, 60 ng/mL for *Bacteroides* species and 500 ng/mL for *Ureaplasma urealyticum*. A single 1 g dose does not maintain amniotic fluid concentrations above the highest of these thresholds across a seven-day latency. That criterion, however, presupposes that maintenance of concentrations above an MIC-like threshold is the pharmacodynamic index of interest. For macrolides, antimicrobial activity is primarily exposure-dependent and is better characterised by the ratio of the area under the concentration–time curve to the minimum inhibitory concentration (AUC/MIC) than by time above MIC [26]. Simulated maintenance above a nominated concentration is therefore a descriptive benchmark rather than evidence that a validated pharmacodynamic target has been attained. To our knowledge, no AUC/MIC target has been clinically validated for *Ureaplasma* species or for the other organisms listed in the PPROM setting. Model-based dose selection is therefore no longer absent from this field. What remains absent is any study linking a modelled dose to an obstetric or neonatal outcome, and the simulations rest on a small observed cohort. The population pharmacokinetic model and the alternative schedules simulated from it are therefore hypothesis-generating: they do not constitute dose recommendations, and they cannot establish an optimal clinical dosing regimen.

A retrospective cohort of 296 pregnancies found no difference in latency between single-dose and five-day azithromycin (5 versus 4 days, *p* = 0.641) but significantly more histological chorioamnionitis with the single dose (62.6% versus 46.4%, *p* = 0.006) [27]. Together, these suggest that the pharmacokinetic advantage of repeated dosing may have a histological correlate, and that latency is an insensitive endpoint for detecting it. Neither study can determine whether the difference matters clinically.

Route has been examined once, and by accident. When hurricane damage to manufacturing capacity in 2017 curtailed the supply of small-volume intravenous fluid bags, one centre switched to an entirely oral seven-day regimen of azithromycin plus amoxicillin. Comparison of 37 women managed orally with 79 managed with the standard two-day intravenous phase followed by five oral days showed a mean latency difference of −0.15 days (95% CI −4.87 to 4.58), with composite maternal infection RR 0.43 (95% CI 0.05–3.53) and composite neonatal infection RR 0.43 (95% CI 0.05–3.52) [28]. The confidence intervals are wide enough to accommodate substantial harm or benefit in either direction; the authors concluded, appropriately, that an oral-only strategy is worthy of further study. That conclusion has not yet been acted on. The question matters practically, because the initial intravenous phase is a principal reason for inpatient admission in some systems, and because oral therapy would simplify management in settings where intravenous access and infusion capacity are limited.

Pharmacokinetic data can explain why a regimen might work and can generate dosing hypotheses, but pharmacological plausibility is not demonstrated clinical benefit.

## 6. Duration: Why Seven Days?

### 6.1. The Origin of the Number

The seven-day course exists because the NICHD protocol combined 48 h of intravenous therapy with five days of oral therapy [11]; ORACLE I used up to ten days or until delivery [12]. Neither figure came from a duration-ranging exercise; both were pragmatic design decisions. That two different durations were embedded in the two positive trials, and that both survive in different guidelines today, is itself informative: the field inherited two numbers rather than converging on one.

### 6.2. Shorter Courses

Two randomised trials from 2003 examined shortening, and both are routinely misread. Lewis et al. enrolled 84 women across three centres in an intention-to-treat trial, allocating 42 to three days and 42 to seven days of ampicillin–sulbactam 3 g intravenously every six hours [29]. Latency did not differ (214 ± 225 h with three days versus 229 ± 218 h with seven), no other parameter differed, and significantly more women completed the shorter course (80.1% versus 47.6%, *p* = 0.003; the completion figure of 80.1% is as published and is not an exact proportion of 42). The authors concluded that more patients would be needed to exclude a type II error. Segel et al. randomised 48 women to three or seven days of ampicillin, taking achievement of seven-day latency as the primary outcome; the relative risk was 0.83 (95% CI 0.51–1.38), and chorioamnionitis, postpartum endometritis and composite neonatal morbidity were unchanged [30].

That confidence interval is the point. It is compatible with anything between a 49% reduction and a 38% increase in the chance of reaching seven days’ latency, a range that comfortably includes clinically important harm. Neither trial pre-specified a non-inferiority margin, and neither was powered to exclude one. These two studies constitute the direct randomised evidence for shortening identified in this review. They establish that two small trials did not detect a difference; they do not establish that three days is as good as seven. No adequately powered non-inferiority trial of a shortened course has been performed, and we identified no meta-analysis restricted to duration comparisons; with two small trials there is too little to pool. One national guideline has nonetheless already moved: CNGOF accepts early discontinuation of prophylaxis when the initial vaginal culture is negative, on professional consensus rather than trial evidence [31]. That criterion also assumes that a negative vaginal culture reliably excludes the organisms of interest, which, for the fastidious species most often implicated after membrane rupture, it does not (Section 4.4). The completion-rate difference in the Lewis trial is a reminder that a shorter course is not only a stewardship question but an adherence one.

### 6.3. Longer Courses and Continuation Until Delivery

The evidence for extending therapy is, paradoxically, more recent and more randomised than the evidence for shortening it. A single-centre trial randomised 151 women with PPROM at 22 0/7–33 6/7 weeks to intravenous cefazolin 1 g every 12 h plus oral clarithromycin 500 mg every 12 h for seven days or until delivery [32]. Gestational age at delivery and the rupture-to-delivery interval did not differ. Respiratory distress syndrome (32.8% versus 50.8%, *p* = 0.039) and composite neonatal morbidity (34.4% versus 53.9%, *p* = 0.026) were both lower in the until-delivery arm, and both differences persisted after adjustment for maternal age, twin pregnancy, antenatal corticosteroid use, rupture-to-delivery interval and gestational age at delivery. Infantile neurological outcomes at 12 months did not differ. The trial is small and single-centre, was analysed per protocol and uses agents that no guideline recommends, and the mechanism of a respiratory benefit in the absence of a latency difference is not obvious. It nonetheless remains the larger and better-conducted of two contemporary randomised comparisons of duration, and its direction of effect favoured the longer strategy.

A second randomised comparison was published in 2025. It allocated 110 women with PPROM at 26 0/7–36 6/7 weeks to one of two arms. Both received intravenous ceftriaxone and metronidazole followed by oral clarithromycin, either to complete a seven-day course or continued until delivery. The authors concluded in favour of the seven-day course [33]. That conclusion is not secure. Cervical dilatation at admission differed markedly between arms, the cervix being closed in 76.3% of the seven-day group and 20.0% of the continuation group, and this imbalance raises concern about the effectiveness of the randomisation and allocation procedures. Allocation is described only as simple random sampling. The abstract and the concluding section draw opposite inferences from the same results. Four neonatal deaths, all in infants below 1000 g, occurred in the seven-day arm and none in the continuation arm, yet survival is not discussed as favouring continuation. The trial is prospectively registered (CTRI/2024/08/072969) and must be counted, but its baseline imbalance places it well below the Korean trial in evidential weight.

The two contemporary randomised comparisons therefore disagree, and neither is adequately powered; the disagreement is itself the argument for a properly designed trial.

Observational data are consistent in direction. A retrospective cohort found that extended azithromycin administration was associated with increased latency without an effect on other maternal or neonatal outcomes [89]. The two-era Korean cohort using a broader-spectrum regimen continued until delivery reported the largest latency difference among the studies reviewed here, concentrated in pregnancies with intra-amniotic infection or inflammation [72]. Both designs are vulnerable to secular confounding and to indication bias, and neither can separate the effect of the agents from the effect of the duration.

### 6.4. What Happens After Day Seven

The practical significance of the duration question is easily underestimated, because most expectantly managed PPROM pregnancies outlive their antibiotic course. In a contemporary cohort of pregnancies with PPROM before 32 weeks, median latency was 16 days (IQR 4–27), and only 29–35% of women delivered within seven days across gestational strata [35]. For most women, therefore, therapy stops while the membranes remain ruptured and the pregnancy continues, sometimes for weeks.

What should be done in that interval is unknown. We identified no trial evaluating a repeat course, restarting antibiotics on the basis of duration of rupture alone or continuing prophylaxis indefinitely. Practice varies accordingly. It is worth stating the underlying principle plainly: prolonged membrane rupture is not, by itself, an indication for prolonged antimicrobial therapy. Antibiotics given after the initial course would need to be justified either by evidence of continuing benefit—which does not exist—or by a new clinical indication, in which case the treatment is no longer prophylaxis. Where a woman develops clinical evidence of intra-amniotic infection, the correct response is therapeutic antibiotics and delivery, not an extension of latency prophylaxis [5].

Professional society guidance is cautious in the same direction, with SMFM advising against prolonged or repeated courses on stewardship grounds [34]—a reasonable default in the absence of evidence, but a default, not a conclusion. In practical terms, seven days is a historically successful protocol duration that became standard practice; shortening it has not been adequately tested, and the more methodologically interpretable of the two contemporary randomised comparisons raises a legitimate question about whether stopping at seven days is optimal. That question justifies further trials; it does not justify adopting prolonged therapy as a new standard.

## 7. Maternal and Obstetric Consequences of Regimen Selection

For the obstetrician, the outcomes that determine management are latency, intra-amniotic infection, maternal infectious morbidity and the decision to continue or abandon expectant management. Antibiotics as a class influence the first two, by the margins set out in Section 1 [4]. Whether one regimen influences them more than another is far less clear, and the two available comparative signals point in opposite directions of confidence: macrolide monotherapy appears inferior to combination therapy in observational data [13], while azithromycin and erythromycin appear indistinguishable in latency terms with a possible advantage for azithromycin with respect to chorioamnionitis [15]. The first of these signals comes from a retrospective cohort in which macrolide monotherapy was used predominantly in women with reported penicillin allergy; confounding by indication therefore precludes attributing the difference to the regimen itself.

Outcome definition is an under-recognised source of noise in these comparisons. The NICHD workshop that introduced the “Triple I” terminology sought to separate isolated maternal fever from suspected and confirmed intrauterine inflammation or infection [90]. When those criteria were tested in 339 febrile women, sensitivity for an infectious outcome was 71.4% (95% CI 61.4–80.1) and specificity 40.5% (95% CI 33.6–47.8) [91]. An endpoint with that measurement performance will dilute genuine differences between regimens, particularly in small studies. Future comparative trials should specify the infection endpoint precisely and pair clinical assessment with placental histological assessment, since the two do not track each other closely: in the azithromycin dosing cohort described above, latency was unchanged while histological chorioamnionitis differed by 16 percentage points [27].

Maternal safety considerations are modest but not negligible. Erythromycin’s gastrointestinal intolerance is a recognised reason for substitution, although no PPROM-specific study has quantified discontinuation rates—a small but real evidence gap. Macrolide exposure in the first trimester has been associated with major malformations (27.65 versus 17.65 per 1000; adjusted risk ratio 1.55) and cardiovascular malformations (10.60 versus 6.61 per 1000; adjusted risk ratio 1.62) compared with penicillins within a UK cohort of 104,605 children [92]. That signal concerns organogenesis and does not apply to latency therapy given in the second or third trimester, but it is relevant to counselling and to the wider question of macrolide safety in pregnancy.

Finally, regimen selection operates within a management strategy whose own risks are substantial: in a cohort of 234 women with PPROM at 22–33 weeks, 36% experienced at least one significant complication, 45% of them within the first three days [93], and abruption and cord prolapse are both more frequent at earlier gestations [94,95]. Antibiotic choice does not modify these risks and should not dominate a decision that also involves surveillance intensity, place of care and timing of delivery.

## 8. Foetal and Neonatal Consequences

Neonatal outcomes matter here because they are the principal justification for prolonging a pregnancy at all. The pooled randomised evidence shows reductions in neonatal infection (RR 0.67, 95% CI 0.52–0.85), surfactant use (RR 0.83, 95% CI 0.72–0.96), oxygen therapy (RR 0.88, 95% CI 0.81–0.96) and abnormal cerebral ultrasound findings (RR 0.81, 95% CI 0.68–0.98), without a significant reduction in perinatal mortality [4]. The last point is often omitted when the benefit of latency antibiotics is summarised, and it should not be: the demonstrated effect is on morbidity, not survival.

Whether these benefits are sensitive to regimen is largely untested. Erythromycin alone reduced neonatal sepsis in the network meta-analysis (RR 0.74, 95% CI 0.56–0.97), but no regimen outperformed placebo for death or necrotising enterocolitis, and none was consistently superior [14]. The clearest regimen-specific neonatal effect in the literature is a harm: the excess of necrotising enterocolitis with co-amoxiclav [4]. Long-term follow-up of the PPROM cohort of ORACLE I found no difference in functional impairment at seven years with either antibiotic [57]; the cerebral palsy signal that is sometimes attributed to latency antibiotics arose in the intact-membrane cohort and does not apply here [56].

The most important contemporary consideration is that the neonatal infections these regimens are intended to prevent have changed. In the NICHD Neonatal Research Network surveillance of 2015–2017, early-onset sepsis occurred in 1.08 per 1000 live births (95% CI 0.95–1.23), and *Escherichia coli* accounted for 36.6% of cases against 30.2% for GBS, with the highest rates among infants born between 22 and 28 weeks [96]. Ampicillin resistance is common in this population: among neonatal bacteraemias in the Canadian PPROM cohort, 11 of 19 isolates (58%) were ampicillin-resistant [13], and in a Chinese single-centre series *E. coli* isolated from preterm infants was ampicillin-resistant in 93.3% of cases versus 48.4% in term infants [19]. Intrapartum antibiotic exposure has been identified as an independent risk factor for ampicillin-resistant *E. coli* sepsis [97]. These estimates are not interchangeable. They come from different centres, populations and calendar periods [98], and reported ampicillin resistance in neonatal *E. coli* varies with local antimicrobial-resistance epidemiology, with gestational age [19] and with prior antibiotic exposure and the era in which a series was assembled [97]; the Canadian figure rests on 19 isolates from five centres [13]. Taken together they indicate that ampicillin resistance is a serious and plausibly growing consideration in some settings, but they do not provide a resistance rate that can be transferred to any given PPROM population. None of this establishes that the standard regimen has lost its effect—the effect was never predominantly attributable to eradication of neonatal pathogens—but it does mean that the microbiological assumptions underpinning a 1997 protocol cannot simply be assumed to hold in 2026.

## 9. Antimicrobial Resistance, Microbiome and Stewardship: What Matters to the Obstetrician

Three practical implications follow from the resistance and microbiome literature, separate from the much larger mechanistic body that does not bear on obstetric decisions.

First, resistance is relevant to regimen selection because it may alter effectiveness, not merely ecology. Given the resistance figures set out in Section 8 and the centre-, population- and era-specific character of those figures, the question of whether an alternative β-lactam performs better is a clinical question, and the observation that a cefuroxime-based protocol was associated with fewer ampicillin-resistant *Enterobacteriaceae* and less chorioamnionitis than an ampicillin-based one [71] is a legitimate prompt for a trial rather than a reason for unilateral change. Because resistance prevalence is a local and changing variable, the case for testing an alternative backbone is a case for prospective evaluation informed by contemporary local epidemiology, not a general conclusion that ampicillin is microbiologically inadequate.

Second, unnecessary exposure is a real cost falling on both mother and infant. Every additional day beyond the evidence-supported course, every repeat course prescribed for prolonged rupture alone, and every broad-spectrum substitution made without comparative evidence adds exposure without demonstrated benefit. That is the reasoning behind current professional society caution about prolonged or repeated courses in the previable and periviable window [34].

Third, microbiome effects are demonstrable but their clinical meaning is not. Intrapartum penicillin prophylaxis for GBS colonisation was followed by reduced relative abundance of *Bifidobacterium longum* at one month (12.0% versus 23.8%, *p* = 0.02) and at one year (9.5% versus 11.4%, *p* = 0.044), with a proinflammatory T-helper profile, but clinical outcomes including weight, infections and hospitalisation did not differ in the first year [99]. No microbiome data specific to multi-day antenatal latency regimens were identified, which is itself an evidence gap; the intrapartum prophylaxis signal is offered as an illustration of the general phenomenon rather than as latency-specific evidence. Such findings are a reason to avoid unnecessary exposure. They are not a reason to withhold a therapy with randomised evidence of neonatal benefit, nor to justify shortening a course that has not been shown to be safely shortenable.

We identified no antimicrobial stewardship guidance addressing latency regimens specifically, as distinct from general obstetric stewardship principles. Stewardship principles in this setting are therefore applied by extrapolation from general frameworks, which is itself a gap worth closing.

## 10. Special Obstetric Situations

### 10.1. Reported β-Lactam Allergy

Reported penicillin allergy is the commonest reason for departing from the standard regimen, and it is the situation in which the evidence base is weakest and the potential for harm greatest. Two facts should govern management. The first is that most reported allergy is not allergy: approximately 80–90% of persons reporting a penicillin allergy are not truly allergic, and allergy testing is safe in pregnancy [6]. The second is that simply deleting the β-lactam is not a neutral act. In the Canadian multicentre retrospective cohort, macrolide monotherapy—used predominantly in women with reported allergy—was associated with shorter latency, more chorioamnionitis and more intraventricular haemorrhage than a β-lactam–macrolide combination [13]. That comparison is observational and confounded by indication: the reported allergy that prompted macrolide monotherapy also defines the exposed group, so the association cannot establish that combination therapy is causally superior. It does, however, argue against assuming that deletion of the β-lactam is outcome-neutral.

A structured approach follows. Where the history is low-risk (isolated gastrointestinal symptoms, non-urticarial rash without systemic features, pruritus without rash, family history alone or no recollection of the reaction), a cephalosporin is appropriate; cross-reactivity is approximately 4.3% with first- and second-generation cephalosporins and below 1% with third- and fourth-generation agents [6]. Where the history suggests an IgE-mediated reaction, allergy evaluation during pregnancy should be considered, and the regimen should retain an agent active against GBS and Gram-negative organisms rather than default to a macrolide alone. A secondary analysis of 949 pregnancies enrolled in a randomised trial of antenatal magnesium sulphate found that non-penicillin regimens gave comparable latency and chorioamnionitis rates but differing patterns of maternal and neonatal morbidity [20]; substitution has consequences rather than being a neutral swap. ACOG is explicit that, although no alternative regimen has been well studied, it may be reasonable to replace the β-lactam with another agent effective against GBS, the choice depending on the severity of the reported reaction and on GBS susceptibility where known [1]. Other guidelines address erythromycin intolerance but specify no alternative for true β-lactam allergy [87,88], and FIGO’s stated alternative runs the other way, substituting a β-lactam when the macrolide cannot be given [100].

### 10.2. Group B Streptococcal Colonisation

Latency antibiotics and intrapartum GBS prophylaxis have distinct indications and must not be conflated. If expectant management is planned, a GBS culture should be obtained at presentation and the latency regimen should include an agent active against GBS [6]. ACOG states directly that “latency antibiotics that include ampicillin given in the setting of preterm prelabour rupture of membranes are adequate for GBS prophylaxis” and cites prospective evidence that GBS was no longer recoverable from vaginal–rectal swabs three days after intravenous treatment directed at the organism was started [6]. Extending latency therapy beyond that point solely to maintain GBS coverage is therefore unlikely to be necessary. Intrapartum prophylaxis remains indicated at the time of delivery for eligible women regardless of earlier antibiotic exposure [1,6]. Evidence on the duration of GBS-directed prophylaxis specifically in women with PPROM who are not in labour is limited [101].

GBS status may nonetheless modify obstetric strategy rather than antibiotic choice. In a secondary analysis from the PPROMEXIL trials of 723 women with PPROM at 34–37 weeks, early-onset neonatal sepsis occurred in 15.2% of the GBS-positive women managed expectantly versus 1.8% with immediate delivery, whereas the GBS-negative women showed no meaningful difference (2.6% versus 2.9%) [102]. This is a subgroup finding from trials not powered for it, and it is hypothesis-generating. It illustrates, however, that GBS colonisation is more likely to change the decision about whether to continue expectant management than the decision about which antibiotic to prescribe.

### 10.3. Previable and Periviable PPROM

Whether evidence derived from PPROM at 24–32 weeks can be extrapolated below the threshold of viability is one of the least resolved questions in this field. SMFM recommends antibiotics for those choosing expectant management at or after 24 0/7 weeks (GRADE 1B), and states that antibiotics can be considered between 20 0/7 and 23 6/7 weeks (GRADE 2C). The same document notes explicitly that “the optimal antibiotic type, dose, and timing of administration after previable and periviable PPROM are unknown” and cautions “against prolonged or repeated antibiotic courses beyond what would be used for PPROM at later gestational ages” [34]. The direct evidence is thin: in a retrospective cohort of 46 pregnancies with rupture before 23 weeks, median latency did not differ significantly with antibiotic receipt (1.0 versus 0.6 weeks, *p* = 0.27) [39]—a small study with a null result, not a demonstration that antibiotics are ineffective before viability.

The context is one of high maternal risk and uncertain neonatal benefit. Survival to discharge varies steeply with gestational age at rupture—12.2% for rupture at 12–19 6/7 weeks versus 43.8% at 20–23 6/7 weeks in a cohort of 130 pregnancies (*p* < 0.001) [36]—and differs across series with differing case mix [103,104,105]. A meta-analysis of five studies (535 pregnancies) compared expectant management with immediate delivery. Expectant management carried an absolute excess of maternal sepsis of 4 percentage points (risk difference 4%, 95% CI 2–7) and of chorioamnionitis of 30 percentage points (*p* < 0.01). Neonatal survival was 39% with expectant management and 0% after immediate delivery (*p* < 0.01) [37]. The 0% figure is structurally determined rather than pharmacological: in this setting immediate delivery means delivery at a previable gestational age, at which survival is not expected whatever antimicrobial therapy is given. It quantifies the consequence of the gestational age at which delivery occurs and must not be read as a treatment effect, nor as evidence that antibiotic-supported expectant management caused the survival difference; the two strategies also differ systematically in the pregnancies for which each was chosen. In a three-centre cohort of 208 pregnancies, composite maternal morbidity was 60.2% with expectant management versus 33.0% after termination (adjusted OR 3.47, 95% CI 1.52–7.93), and only 15.7% of women choosing expectant management both avoided morbidity and had a neonate surviving to discharge [38]. Here the antibiotic decision is a small element within a much larger counselling conversation and should be presented as such.

### 10.4. The Upper Gestational-Age Boundary

Latency antibiotics have no role once expectant management ceases to be advantageous, and randomised evidence at 34–37 weeks is consistent with this. In the PPROMT trial, neonatal sepsis occurred in 2% after immediate delivery versus 3% with expectant management (RR 0.8, 95% CI 0.5–1.3, *p* = 0.37), while respiratory distress (RR 1.6, *p* = 0.008) and mechanical ventilation (RR 1.4, *p* = 0.02) were more frequent after immediate delivery [106]. An earlier Dutch trial found neonatal sepsis in 2.6% versus 4.1% (RR 0.64, 95% CI 0.25–1.6) [107], and an individual participant data meta-analysis of 2563 women found a composite adverse neonatal outcome in 9.6% versus 8.3%, with more respiratory distress after immediate delivery and less chorioamnionitis [108]. ACOG accordingly states that latency antibiotics are not appropriate between 34 0/7 and 36 6/7 weeks [1], although not all societies draw the boundary there [109]. These trials randomised the timing of delivery, not the antibiotic regimen. The upper gestational-age boundary for latency antibiotics is therefore an inference from a randomised comparison of management strategies, not a directly tested parameter. It is nonetheless the best-supported boundary in this field, a fact that indicates how thin the direct evidence is elsewhere.

### 10.5. Prolonged Latency and Suspected Infection

Two situations recur once the initial course is complete. The first is uncomplicated prolonged latency, discussed in Section 6.4: prolonged rupture alone does not justify further antimicrobial therapy. The second is a new clinical concern for infection. Here the distinction between prophylaxis and treatment becomes decisive. Suspected or confirmed intra-amniotic infection is an indication for therapeutic antibiotics and for delivery, not for intensification of latency prophylaxis, and the regimens differ accordingly [5,90]. Conflating the two leads to two characteristic errors: treating established infection with a prophylactic regimen and continuing expectant management in a woman who requires delivery.

## 11. What Current Guidelines Actually Agree on

Major societies agree on four things: that antibiotics should be offered during expectant management of preterm PPROM, that co-amoxiclav should not be used, that the intervention is prophylactic rather than therapeutic and that intrapartum GBS prophylaxis is a separate indication. They disagree, quietly but substantively, on almost every operational detail. Contemporary international guideline recommendations are summarised in Table S2.

The disagreements are structured. Guidance descending from the NICHD trial specifies a β-lactam–macrolide combination for seven days with an intravenous lead-in [1,34]. Guidance descending from ORACLE I specifies oral erythromycin for up to ten days or until established labour [86,87,88]. RANZCOG offers both, including a hybrid in which a seven-day amoxicillin or ampicillin course is combined with ten days of erythromycin [110]. German-language guidance specifies intravenous ampicillin for two days followed by five days of oral amoxicillin together with oral azithromycin, while stating that no clear recommendation can be made for the dose or duration of the azithromycin component [109]. FIGO recommends erythromycin 250 mg every six hours for up to ten days, with a β-lactam alone—ampicillin followed by amoxicillin—as the alternative where erythromycin is contraindicated [100]. The French national guidance departs furthest, permitting amoxicillin, a third-generation cephalosporin or erythromycin as monotherapy for seven days (Grade C), specifying no doses, and being the only guideline in Table S2 that allows antibiotics to be stopped early when the initial vaginal culture is negative [31]. The Canadian national guideline published in 2022, the first to cover PPROM management as a whole rather than antibiotic therapy alone, applies to all patients with rupture before 37 weeks and grades its recommendations using GRADE. It accepts either a macrolide alone, with group B streptococcal cover for two days where GBS status is positive or unknown, or a combination of ampicillin or amoxicillin with a macrolide irrespective of GBS status; it names erythromycin, azithromycin and clarithromycin as acceptable macrolides, specifies no doses, and states that no data support treatment beyond ten days [111]. It is included in Table S2.

These are not trivial variations. They differ in spectrum, in route, in total antimicrobial exposure and in duration. Despite these operational differences, most contemporary recommendations remain heavily anchored in the same landmark randomised evidence, particularly the NICHD and ORACLE I trials, interpreted differently. Guideline consensus on the principle therefore coexists with guideline divergence on the specifics, and neither the consensus nor the divergence reflects head-to-head comparative evidence.

Three further observations are worth recording. The upper gestational-age boundary is not agreed: against the ACOG position set out above, the German-language guideline advises the same antibiotic management between 34 0/7 and 36 6/7 weeks as below 34 weeks [109]. Several guidelines address intolerance of erythromycin but not true β-lactam allergy, leaving the commonest deviation from standard therapy without specific guidance [87,88,100]; ACOG is the exception, and even it names no specific agent or dose [1]. And the WHO recommendation on the agent of choice is graded as conditional on moderate-quality evidence, while its recommendation against co-amoxiclav is graded strong [88]—an accurate reflection of the fact that the negative recommendation rests on firmer ground than the positive one.

## 12. Agent, Dose and Duration: What Remains Unanswered?

**Figure 2:**
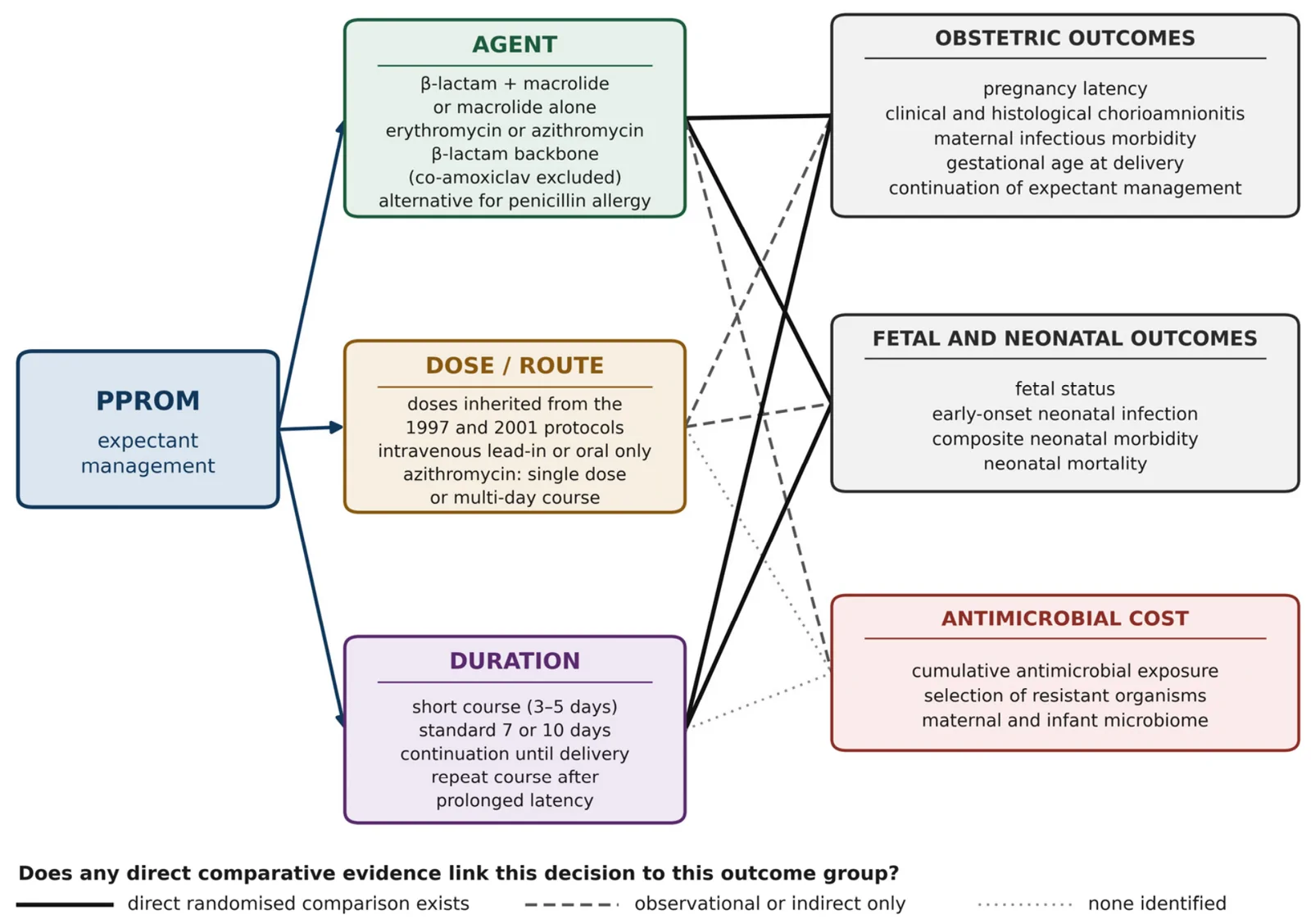
Agent, dose and duration: the three unresolved dimensions of latency antibiotic therapy in PPROM. The central node represents the clinical decision point—a pregnancy with preterm prelabour rupture of membranes (PPROM) entering expectant management. Three therapeutic decisions radiate from it. Agent encompasses the choice between a β-lactam–macrolide combination and macrolide monotherapy, the choice of macrolide, the choice of β-lactam backbone with co-amoxiclav excluded, and the substitution required by reported penicillin allergy. Dose and route encompasses the doses inherited from the 1997 and 2001 protocols, the intravenous lead-in versus an entirely oral strategy, and single-dose versus multi-day azithromycin. Duration encompasses short courses, the standard seven- or ten-day course, continuation until delivery, and repeat courses when latency is prolonged. Each decision connects to the outcomes that determine obstetric management, to fetal and neonatal outcomes, and to the antimicrobial cost of therapy. Line style records whether direct comparative evidence links each decision to each outcome group, not whether that evidence is adequate: solid, at least one randomised trial directly compares options within that dimension and reports that outcome group; dashed, only observational or indirect (network) comparison; dotted, no comparative evidence identified. Solid lines should not be read as adequacy—the underlying trials are small, heterogeneous in regimen and gestational age, and were not designed to identify an optimal configuration. The figure’s point is that the dose and route dimension rests on no randomised comparison using clinical outcome endpoints, although randomised pharmacokinetic evidence exists, and that no dimension has comparative evidence bearing on cumulative antimicrobial exposure or resistance. PPROM, preterm prelabour rupture of membranes.

Figure 2 sets the three decisions against the outcomes they are intended to influence. The remaining evidence gaps and the corresponding research priorities are set out, domain by domain, in Table 1.

### 12.1. Agent

No adequately powered randomised trial has shown any specific regimen to be superior to any other for the comparisons that matter most in practice. Three questions are open. Whether a β-lactam–macrolide combination outperforms macrolide monotherapy is supported observationally [13]—in a retrospective cohort in which macrolide monotherapy was used predominantly in women with reported penicillin allergy, so that confounding by indication precludes causal inference—and by indirect comparison [14], but no direct randomised comparison was identified. Whether azithromycin can replace erythromycin without loss of efficacy rests on five observational cohorts and their meta-analysis [15], together with one small open-label randomised comparison [17]. The pooled latency estimate is a tight null, but no adequately powered trial with a pre-specified non-inferiority margin has been performed. Whether a broader-spectrum β-lactam improves outcomes given the ampicillin resistance reported in some neonatal series has been tested in two small randomised trials from a single research group [69,70] and supported by non-randomised data [71,72]. Those resistance estimates are centre-, population- and era-specific and should not be generalised to all PPROM populations. The signal is consistent in direction and consistently underpowered.

### 12.2. Dose and Route

No PPROM trial has compared two doses of the same agent with clinical endpoints. Azithromycin dosing has been compared pharmacokinetically in six women [24], modelled by population pharmacokinetics with dose simulation [25], and compared retrospectively for histological outcomes [27]. Those simulations rest on the same six observations, are indexed to concentration thresholds rather than to a validated AUC/MIC target and are hypothesis-generating rather than dose recommendations. No randomised dosing comparison identified has used clinical outcome endpoints. Route has been compared once, opportunistically, in 116 women, with confidence intervals wide enough to include clinically important differences either way [28]. Whether the intravenous phase contributes to the benefit or is simply how the original protocol happened to be written, is unknown, and the answer bears directly on admission practice and on resource-limited settings.

### 12.3. Duration

Seven days and ten days are both protocol-derived durations, and neither was selected through duration-ranging optimisation. Shortening has been examined in two small randomised trials whose null results do not establish equivalence; the wider confidence interval still admits a 49% reduction in the chance of reaching seven days’ latency [29,30]. Extension to delivery has been examined in two small randomised trials that reached opposite conclusions [32,33], and in observational cohorts favouring the longer course [72,89]. The interval after the course ends, where most expectantly managed pregnancies spend most of their latency [35], has not been studied.

### 12.4. Cross-Cutting Uncertainties

Three issues cut across all three domains. Gestational age is the most obvious. The NICHD trial enrolled women between 24 and 32 weeks [11], yet the regimen is applied from previability to 34 weeks, and no comparison identified has been stratified by gestational age at rupture, despite marked differences in latency and competing risks across that range [35,36]. Outcome definition compounds the problem: heterogeneous and imperfectly performing definitions of intra-amniotic infection [91] make small comparative studies difficult to interpret, and microbiological ascertainment varies in the same way, because culture-based and molecular methods do not detect the same organisms. Least tractable is contemporaneity. The evidence underpinning current practice predates universal antenatal corticosteroid use, current viability thresholds, current neonatal intensive care and the emergence of *E. coli* as the dominant early-onset pathogen [96]. None of this invalidates the historical trials, but the specific regimen has not been re-evaluated under contemporary conditions.

## 13. Priorities for Future Obstetric Research

Four pragmatic comparative trials appear particularly feasible and clinically informative.

The first concerns one of the largest unexplained divergences between international guidelines and the divergence with the strongest observational signal of a difference: a pragmatic randomised comparison of a β-lactam–macrolide combination against macrolide monotherapy [13]. That signal arises from a retrospective cohort in which macrolide monotherapy was used predominantly in women with reported penicillin allergy; confounding by indication is precisely why randomisation, rather than a further observational comparison, is required to determine whether the difference is causal. Clinical equipoise is plausible because both strategies are currently guideline-supported in different jurisdictions.

The second is a properly designed non-inferiority trial of azithromycin against erythromycin, with a latency margin agreed in advance to be obstetrically acceptable. Substitution has already happened in practice; the evidence has not caught up. A trial with a respiratory rather than an infectious primary endpoint is recruiting in extremely preterm PPROM [18], and its design makes a broader point: the endpoint should be the outcome that actually drives the obstetric decision.

The third is a duration trial. Two small contemporary randomised comparisons disagree, one favouring continuation until delivery [32] and one favouring a fixed course [33], while stewardship pressure points towards the shorter option. A three-arm comparison of a short course, the standard seven-day course and continuation until delivery would resolve a question relevant to routine expectant management of PPROM. Such a trial should be powered for a composite of clinically meaningful neonatal morbidity, should include maternal infectious morbidity and should collect resistance endpoints prospectively rather than as an afterthought.

The fourth is an adequately powered comparison of β-lactam backbones, ampicillin against a second- or third-generation cephalosporin. Two small randomised trials from a single Israeli group have produced signals favouring the cephalosporin-based backbone, one for latency and the other for maternal infectious morbidity [69,70], while the rationale is reinforced by the frequency of ampicillin resistance among neonatal isolates after PPROM [13,19,96], although those series differ in centre, population, gestational age and calendar period, so they define a question to be answered prospectively in contemporary populations with documented local resistance epidemiology rather than establishing a general microbiological inadequacy of ampicillin. What is required is external replication with neonatal outcomes and prospectively collected resistance endpoints, not a first test of the hypothesis.

All four proposals are conventional comparative trials, and each would still have to select the doses it compares. Because no dose in current use has been evaluated against a defined pharmacokinetic/pharmacodynamic target in pregnancy, that selection would otherwise be empirical, as it was in the 1990s. Model-informed development offers a way to constrain it before a large trial is mounted. Physiologically based pharmacokinetic (PBPK) modelling can incorporate the gestational-age-dependent changes in renal clearance, volume of distribution and unbound fraction described in Section 5 and predict maternal and, where data permit, foetal, exposure across the 24–32-week window [22]. Population pharmacokinetic (popPK) analysis of prospectively collected samples can quantify between-woman variability in the same population, as has already been attempted for azithromycin on a very small scale [24,25]. Probability-of-target-attainment simulation can then identify the narrow range of doses that plausibly achieves the exposure the chosen index requires—provided that the index and its target are stated explicitly rather than assumed.

Two practical consequences follow. Sparse prospective pharmacokinetic sampling can be embedded within the comparative trials proposed above at modest additional burden, so that exposure data accumulate in the population in which these regimens are actually used rather than in a separate volunteer study. And adaptive designs—in which pre-specified interim analyses permit unpromising dose arms to be dropped, allocation to be updated or the sample size to be re-estimated, with pre-specified adjustment to control the type I error rate [23]—would reduce the number of empirically chosen arms that must be carried to the end of recruitment. Both matter disproportionately here, because PPROM between 24 and 32 weeks is an uncommon and time-critical condition in which recruitment is slow, eligibility windows are short and every avoidable arm lengthens a trial that is already difficult to complete.

None of this substitutes for clinical outcome trials. Target attainment is a surrogate, and a dose that achieves a modelled exposure target has not thereby been shown to prolong latency, reduce chorioamnionitis or improve neonatal outcome. The proper role of PBPK and popPK modelling here is to inform which doses are worth comparing and how the comparison should be structured; whether the regimen so selected benefits women and infants remains a question that only prospective clinical evaluation can answer.

Several design principles apply across all four. Gestational age at rupture should be a pre-specified stratification variable, not a covariate. Infection endpoints should be defined explicitly and, where possible, corroborated histologically. Microbiome and resistance measures belong as secondary or exploratory outcomes, informing rather than replacing clinical results. Microbiological outcomes deserve to be specified rather than left implicit. Where sampling permits, a comparative trial should record the organisms identified before and during treatment, their species distribution, their antimicrobial susceptibility and resistance profiles, the emergence or selection of resistance during therapy where that is measurable and microbiological persistence or eradication. Because culture and molecular assays detect different things (Section 4.4), integrating culture-based susceptibility testing with molecular detection is preferable to either alone wherever that is methodologically feasible. These endpoints are complementary to, and not substitutes for, latency, clinical chorioamnionitis, maternal infectious morbidity, neonatal infection and sepsis and neonatal morbidity and mortality. A trial comparing antimicrobial regimens should ideally answer two questions at once: whether the regimen improves clinically meaningful outcomes and what microbiological and ecological consequences accompany it. Women with reported penicillin allergy, currently the most poorly served group, should be recruited rather than excluded. And trials should be large enough that a null result means something: the recurring failure in this literature has been small studies whose null findings were read as evidence of equivalence.

## 14. Limitations

This review has the limitations inherent to its design. Studies were selected for relevance rather than against pre-specified eligibility criteria, and no risk-of-bias instrument was applied, so the appraisals offered here are structured judgements rather than formal assessments. Screening was not performed independently in duplicate. Two of the older duration trials are available to us only as published abstracts, and their gestational-age ranges and dosing are therefore incompletely characterised in Table S1. The search covered three bibliographic databases, the Cochrane Library and four trial registries. Comparative work published in journals outside the major indexes may therefore be under-represented. One such trial is discussed in Section 4.2; others may not have been retrieved. Guideline documents change between review cycles. The positions summarised in Table S2 are those stated in each document at the last recheck on 20 August 2026, and the review status of each document is recorded in the table as the issuing society reports it. The argument advanced here concerns the strength of comparative evidence, not the direction of clinical benefit; it should not be read as questioning whether latency antibiotics should be offered.

## 15. Conclusions

Latency antibiotic therapy is an established and worthwhile component of expectant management after PPROM. That antibiotics prolong pregnancy, reduce chorioamnionitis and reduce short-term neonatal morbidity has been reproduced across randomised trials and confirmed in the meta-analysis. An obstetrician who prescribes a guideline-concordant latency regimen today is on solid ground.

The configuration of that therapy stands on weaker footing. The core regimens underpinning current guidelines descend from two placebo-controlled trials designed to test antibiotics against placebo rather than against each other, and their doses have not since been compared with alternatives in a PPROM population. Seven days is the sum of a 48-h intravenous phase and a five-day oral phase specified in one of those protocols; ten days is protocol-derived in the same way from the other. Neither number came from a duration-ranging exercise. The most widely adopted recent change in practice, substitution of azithromycin for erythromycin, rests predominantly on observational data and on a single small open-label randomised comparison without a pre-specified non-inferiority margin: pragmatic and guideline-permitted but not confirmed. The two contemporary randomised trials of duration reach opposite conclusions, which is itself the clearest indication that the question remains open.

What follows for practice is calibrated confidence rather than hesitation. Latency antibiotics should continue to be offered in guideline-eligible pregnancies managed expectantly; co-amoxiclav should continue to be avoided; and latency prophylaxis should not be confused with treatment of established intra-amniotic infection or with intrapartum GBS prophylaxis. Observational data also counsel caution before defaulting to macrolide monotherapy in women reporting penicillin allergy, although those data are confounded by indication—allergy status determined the regimen prescribed—and cannot establish that combination therapy is causally superior. Beyond these points, we are following protocols that succeeded rather than protocols that were optimised, and the distinction is not academic: it determines whether a woman who ruptures at 27 weeks receives the best available regimen or merely the historically successful one. Establishing which agent, at which dose and route, and for how long serves women and infants best will require the adequately powered comparative trials that the past twenty-five years have not produced.

## Figures and Tables

**Table 1:** Agent, dose and duration: established evidence versus unanswered questions.

Clinical Question	What is Established	Current Obstetric Practice	Type of Comparative Evidence	What Remains Uncertain	Priority Research Question
Should latency antibiotics be given at all?	Antibiotics prolong pregnancy and reduce chorioamnionitis and neonatal infection; perinatal mortality is not significantly reduced [4,11,12]	Broadly recommended during expectant management before 34 weeks, with society-specific differences in the lower and upper gestational-age boundaries	Direct randomised evidence: multiple placebo-controlled RCTs and a Cochrane meta-analysis	Magnitude of benefit under contemporary neonatal care and corticosteroid use	Contemporary re-estimation of absolute benefit by gestational-age stratum
Combination versus macrolide monotherapy	Combination is associated with longer latency and less chorioamnionitis than macrolide alone in a multicentre retrospective cohort in which macrolide monotherapy was used predominantly in women with reported penicillin allergy [13]; no regimen is consistently superior in network meta-analysis [14]	Divergent by jurisdiction: combination in North America, monotherapy in the UK and WHO guidance	Observational comparative evidence and indirect (network) comparison; no direct randomised comparison; the observational comparison is confounded by indication and cannot establish causality	Whether the difference is causal or reflects confounding by penicillin allergy	Pragmatic RCT of β-lactam + macrolide versus macrolide alone, powered for latency and chorioamnionitis, with prospective microbiological endpoints—organisms identified, species distribution, susceptibility and resistance profiles—alongside the clinical outcomes
Azithromycin versus erythromycin	Latency mean difference 0.07 days (95% CI −0.45 to 0.60); chorioamnionitis pooled OR 0.53 (95% CI 0.39–0.71) [15]; more endometritis and intraventricular haemorrhage with azithromycin in one cohort [16]	Widespread substitution of a single 1 g azithromycin dose	Observational comparative evidence (five cohorts) and one small open-label randomised comparison, not indexed in MEDLINE and without a pre-specified non-inferiority margin [17].	Whether the chorioamnionitis advantage is real or an artefact of pre/post design; whether latency is truly non-inferior	Randomised non-inferiority trial with a pre-specified margin; a phase II RCT with a composite bronchopulmonary dysplasia and mortality endpoint is under way [18]
Choice of β-lactam	Co-amoxiclav increases necrotising enterocolitis (RR 4.72, 95% CI 1.57–14.23) and must be avoided [4]	Commonly used as the β-lactam backbone in combination-based protocols	Direct randomised evidence for the co-amoxiclav harm; for the choice among other β-lactams, two small randomised trials from a single research group across three centres	Whether a cephalosporin outperforms ampicillin given the ampicillin resistance reported in some neonatal series [13,19], whose rates vary by centre, population, gestational age and era	External replication of the two existing trials, with neonatal and resistance endpoints, including prospectively collected organism identification, species distribution and antimicrobial susceptibility profiles, in populations with documented local resistance epidemiology
Management of reported β-lactam allergy	80–90% of reported penicillin allergy is not true allergy; testing is safe in pregnancy; cross-reactivity is approximately 4.3% with first- and second-generation cephalosporins and below 1% with third- and fourth-generation agents [6]	Frequent default to macrolide monotherapy	Observational comparative evidence only, with signals of worse outcomes [13,20], confounded by indication because reported allergy status determined the regimen prescribed	Optimal regimen for genuine IgE-mediated allergy	Trial or prospective registry of allergy delabelling and of cephalosporin-based latency regimens
Dose of each agent	Doses derive from the NICHD and ORACLE protocols [11,12]; pregnancy raises renal clearance, expands the volume of distribution and lowers protein binding, so target attainment cannot be assumed from non-pregnant or historical data [21]	Unchanged since the original protocols	No direct comparative evidence: no dose-comparison trial with clinical endpoints	Whether current doses are adequate, excessive or suboptimal in pregnancy	Model-informed dose optimisation—PBPK [22] and popPK modelling with probability-of-target-attainment simulation against an explicitly stated PK/PD index—to define candidate doses, followed by dose-finding work embedded within a pragmatic trial, using an adaptive design where feasible [23]
Azithromycin single dose versus multi-day course	Multi-day dosing sustains higher amniotic fluid concentrations [24]; a single 1 g dose does not maintain concentrations above the *Ureaplasma* threshold in simulation [25], a susceptibility benchmark rather than a validated pharmacodynamic target, since macrolide activity is AUC/MIC-indexed [26]; single dose is associated with more histological chorioamnionitis [27]	Single 1 g dose predominates	Randomised pharmacokinetic evidence (*n* = 6) and a population pharmacokinetic model with dose simulation derived from the same six women, hypothesis-generating rather than a validated dose recommendation; observational comparative evidence for histological outcomes; no randomised comparison using clinical outcome endpoints	Whether the pharmacokinetic difference has clinical consequences	Adequately powered randomised comparison of azithromycin dosing schedules, with candidate doses selected by target-attainment simulation against an explicitly stated AUC/MIC index
Intravenous versus oral route	Latency mean difference −0.15 days (95% CI −4.87 to 4.58) in one small cohort [28]	Intravenous lead-in of 48 h is standard where combination therapy is used	Observational comparative evidence: a single retrospective historical-control study	Whether the intravenous phase contributes independently to benefit	Non-inferiority trial of an entirely oral regimen
Standard treatment duration	7 days derives from the NICHD protocol; 10 days from ORACLE I	7 or 10 days depending on jurisdiction	Indirect evidence only: no head-to-head comparison of the seven- and ten-day standards	Whether either duration is optimal	Three-arm randomised comparison of 5-day, 7-day and until-delivery strategies
Short-course treatment	Two small randomised trials (*n* = 84 and *n* = 48) detected no difference; the relative risk for achieving 7-day latency was 0.83 (95% CI 0.51–1.38) [29,30]	Rarely used, although CNGOF permits stopping early when the initial vaginal culture is negative [31]	Direct randomised evidence: two small trials, neither with a pre-specified non-inferiority margin	Whether 3–5 days is genuinely non-inferior; existing confidence intervals still include clinically important harm	Adequately powered non-inferiority trial with a stated margin
Prolonged treatment or continuation until delivery	One RCT found lower RDS (32.8% vs. 50.8%) and composite neonatal morbidity (34.4% vs. 53.9%) with continuation until delivery [32]; a second, with marked baseline imbalance, favoured a fixed seven-day course [33]	Not standard; discouraged in the previable window [34]	Direct randomised evidence: two small trials with discordant conclusions, plus observational data	Which of the two discordant trials is closer to the truth; whether the finding replicates with guideline-standard agents; the resistance cost	Multicentre replication with standard agents and resistance endpoints
Repeat courses after prolonged latency	No evidence of benefit; prolonged rupture alone is not an indication	Highly variable	No direct comparative evidence	Whether any subgroup benefits from re-treatment	Observational cohort with protocolised re-treatment criteria, then a trial if a signal emerges
Gestational-age-specific therapy	Latency, survival and complication profiles differ markedly across gestational strata [35,36]	One regimen applied across the whole range	No direct comparative evidence stratified by gestational age at rupture	Whether optimal agent or duration varies with gestational age at rupture	Pre-specified gestational-age stratification in all future trials
Previable and periviable PPROM	Expectant management increases maternal morbidity substantially [37,38]; an antibiotic effect on latency not demonstrated in small cohorts [39]	Antibiotics offered by most clinicians [40]; recommended from 24 weeks, may be considered from 20 weeks [34]	Observational comparative evidence: retrospective cohorts only	Whether antibiotics alter latency or survival before viability, and at what maternal cost	Dedicated trial or prospective multicentre registry in the 20–23 6/7-week window

The fourth column classifies the comparative evidence available for each question by study design, as defined in Section 2.3. It is not a GRADE or certainty rating, and no formal risk-of-bias assessment was undertaken. Abbreviations: AUC, area under the concentration–time curve; CI, confidence interval; MIC, minimum inhibitory concentration; NICHD, Eunice Kennedy Shriver National Institute of Child Health and Human Development; OR, odds ratio; PBPK, physiologically based pharmacokinetic; PK/PD, pharmacokinetic/pharmacodynamic; popPK, population pharmacokinetic; PPROM, preterm prelabour rupture of membranes; RCT, randomised controlled trial; RDS, respiratory distress syndrome; RR, risk ratio.

## Data Availability

No new data were created or analysed in this study. Data sharing is not applicable to this article.

## Supplementary Materials

The following supporting information can be downloaded at: https://www.mdpi.com/article/10.3390/antibiotics15090926/s1. Table S1: Selected landmark and contemporary studies informing latency antibiotic therapy in preterm prelabour rupture of membranes: design, population, regimen, outcomes, limitations, and what each study actually establishes. Studies were selected because they established the therapeutic principle against placebo, compared two or more latency antibiotic strategies against each other, or supplied the pharmacokinetic basis for a dosing decision discussed in the main text. The table is not an exhaustive inventory of the PPROM antibiotic literature. Table S2: Contemporary international guideline recommendations for latency antibiotic therapy in preterm prelabour rupture of membranes: gestational-age range, agent, dose, route, duration, alternatives, allergy and previable guidance, evidence basis, and residual uncertainty.

## Abbreviations

The following abbreviations are used in this manuscript:

Abbreviation	Definition
ACOG	American College of Obstetricians and Gynecologists
AUC	Area under the concentration–time curve
CI	Confidence interval
CNGOF	Collège National des Gynécologues et Obstétriciens Français
DGGG	Deutsche Gesellschaft für Gynäkologie und Geburtshilfe
FIGO	International Federation of Gynecology and Obstetrics
GA	Gestational age
GBS	Group B *Streptococcus*
GRADE	Grading of Recommendations Assessment, Development and Evaluation
HR	Hazard ratio
IQR	Interquartile range
IV	Intravenous
IVH	Intraventricular haemorrhage
MIC	Minimum inhibitory concentration
NEC	Necrotising enterocolitis
NICE	National Institute for Health and Care Excellence
NICHD	Eunice Kennedy Shriver National Institute of Child Health and Human Development
NNH	Number needed to harm
OEGGG	Österreichische Gesellschaft für Gynäkologie und Geburtshilfe
OR	Odds ratio
PBPK	Physiologically based pharmacokinetic
PK/PD	Pharmacokinetic/pharmacodynamic
popPK	Population pharmacokinetic
PPROM	Preterm prelabour rupture of membranes
RANZCOG	Royal Australian and New Zealand College of Obstetricians and Gynaecologists
RCOG	Royal College of Obstetricians and Gynaecologists
RCT	Randomised controlled trial
RDS	Respiratory distress syndrome
RR	Risk ratio
SGGG	Schweizerische Gesellschaft für Gynäkologie und Geburtshilfe
SMFM	Society for Maternal–Fetal Medicine
SOGC	Society of Obstetricians and Gynaecologists of Canada
WHO	World Health Organization
