# Supplementary material for: Agent, Dose and Duration: The Unanswered Questions Behind Latency Antibiotic Therapy for Preterm Prelabour Rupture of Membranes
Source: Antibiotics (Basel). 2026 Sep 18;15(9):926. doi: 10.3390/antibiotics15090926 (PMC13603082; doi:10.3390/antibiotics15090926)
Supplement: Supplementary file 1 [file antibiotics-15-00926-s001.zip › antibiotics-4563760-supplementary.pdf]

Supplementary Materials

Agent, Dose and Duration: The Unanswered Questions Behind Latency Antibiotic Therapy for Preterm Prelabour Rupture of Membranes

Reference numbers used in these Supplementary Tables correspond to the numbered reference list of the main manuscript; no separate reference numbering is used.

Table S1. Selected landmark and contemporary studies informing latency antibiotic therapy in preterm prelabour rupture of membranes: design, population, regimen, outcomes, limitations, and what each study actually establishes. Studies were selected because they established the therapeutic principle against placebo, compared two or more latency antibiotic strategies against each other, or supplied the pharmacokinetic basis for a dosing decision discussed in the main text. The table is not an exhaustive inventory of the PPROM antibiotic literature.

Study (year)	Design	Population / GA / N	Regimen: agent, dose, route, duration	Comparator	Main obstetric outcomes	Main neonatal outcomes	Principal limitation	What the study actually establishes
Mercer et al. (1997) [11]	Multicentre, double-blind, placebo-controlled RCT	PPROM 24 0/7–32 0/7 wk; n = 614	IV ampicillin 2 g q6h + erythromycin 250 mg q6h × 48 h, then oral amoxicillin 250 mg q8h + erythromycin base 333 mg q8h × 5 d (7 d total)	Placebo	Significant pregnancy prolongation in the GBS-negative cohort	Composite morbidity 44.1% vs 52.9% (p = 0.04); RDS 40.5% vs 48.7% (p = 0.04); NEC 2.3% vs 5.8% (p = 0.03); GBS-negative cohort: sepsis 8.4% vs 15.6% (p = 0.01), pneumonia 2.9% vs 7.0% (p = 0.04)	Corticosteroids and tocolysis prohibited after randomisation; narrow GA window; no active comparator	That this specific 7-day β-lactam–macrolide regimen is superior to placebo in PPROM at 24–32 wk. It does not establish that the agents, doses or 7-day duration are optimal
ORACLE I (2001) [12]	Multicentre, double-blind, placebo-controlled 2 × 2 factorial RCT	PPROM < 37 wk; n = 4826	Erythromycin 250 mg qds and/or co-amoxiclav 325 mg qds, orally, up to 10 d or until delivery	Placebo	Not the primary endpoint	Composite death/chronic lung disease/major cerebral abnormality with erythromycin: 12.7% vs 15.2% in all 2415 infants (p = 0.08) and 11.2% vs 14.4% in the 2260 singletons (p = 0.02); NEC excess with co-amoxiclav	Broad GA range including late preterm; the significant primary-outcome benefit is confined to the singleton subgroup; no β-lactam–macrolide combination arm comparable to the NICHD regimen	That allocation to erythromycin was associated with improvement in the composite neonatal outcome in the singleton subgroup, and that co-amoxiclav should be avoided because of the excess of necrotising enterocolitis. The 2 × 2 factorial design yields a marginal comparison pooled across the co-amoxiclav strata; it does not constitute a direct comparison of erythromycin monotherapy with the NICHD combination regimen
ORACLE II (2001) [55]	Multicentre, double-blind, placebo-controlled 2 × 2 factorial RCT	Spontaneous preterm labour, **intact** membranes; n = 6295	As ORACLE I	Placebo	No benefit	No regimen reduced the composite outcome versus placebo	Different population; included here only for contrast	That the PPROM benefit does not generalise to intact membranes — the indication is membrane rupture, not preterm labour
ORACLE Children Study, ORACLE I cohort (2008) [57]	7-year follow-up of RCT	3298 children of ORACLE I participants	As ORACLE I	Placebo	—	Any functional impairment 38.3% vs 40.4% with erythromycin (OR 0.91, 95% CI 0.79–1.05); 40.6% vs 38.1% with co-amoxiclav (OR 1.11, 95% CI 0.96–1.28)	75% follow-up; parent-reported outcome	Long-term reassurance for antibiotic exposure in the PPROM population

Study (year)	Design	Population / GA / N	Regimen: agent, dose, route, duration	Comparator	Main obstetric outcomes	Main neonatal outcomes	Principal limitation	What the study actually establishes
ORACLE Children Study, ORACLE II cohort (2008) [56]	7-year follow-up of RCT	3196 children of ORACLE II participants	As ORACLE II	Placebo	—	Functional impairment 42.3% vs 38.3% (OR 1.18, 95% CI 1.02–1.37); cerebral palsy 3.3% vs 1.7% with erythromycin (OR 1.93, 95% CI 1.21–3.09) and 3.2% vs 1.9% with co-amoxiclav (OR 1.69, 95% CI 1.07–2.67); NNH 64 and 79	Intact-membrane population; 71% follow-up	That antibiotics given for preterm labour with intact membranes may cause long-term harm. This finding must **not** be transferred to PPROM
Cochrane review (2013) [4]	Systematic review and meta-analysis, 22 RCTs	6872 women and infants	Various	Placebo/no treatment	Chorioamnionitis RR 0.66 (0.46–0.96); birth < 48 h RR 0.71 (0.58–0.87); birth < 7 d RR 0.79 (0.71–0.89)	Neonatal infection RR 0.67 (0.52–0.85); surfactant RR 0.83 (0.72–0.96); oxygen RR 0.88 (0.81–0.96); abnormal cerebral ultrasound RR 0.81 (0.68–0.98); co-amoxiclav and NEC RR 4.72 (1.57–14.23); perinatal mortality not significantly reduced	Last substantive update 2013; pooled across heterogeneous regimens; predates azithromycin-era data	That antibiotics as a class benefit PPROM pregnancies, and that co-amoxiclav is harmful. It does not identify an optimal regimen
Chatzakis et al. (2020) [14]	Network meta-analysis, 20 RCTs, 15 regimens	7169 participants	Various	Placebo/no treatment and each other	Chorioamnionitis reduced by clindamycin + gentamicin RR 0.19 (0.05–0.83), penicillin RR 0.31 (0.16–0.60), ampicillin RR 0.52 (0.34–0.81), erythromycin + amoxicillin RR 0.71 (0.55–0.92)	Erythromycin alone reduced neonatal sepsis RR 0.74 (0.56–0.97); no regimen reduced death or NEC versus placebo	Indirect comparisons; evidence quality moderate to very low; predates azithromycin data	That no regimen is clearly and consistently superior on current randomised evidence
Lewis et al. (2003) [29]	Multicentre (3 sites), prospective, randomised, intention-to-treat trial, described by its authors as preliminary	PPROM; n = 84 (42 per arm); GA range not stated in the published abstract	Ampicillin–sulbactam 3 g IV q6h for 3 days	Same regimen for 7 days	Latency 214 ± 225 h vs 229 ± 218 h (no difference); significantly more women completed the 3-day course (80.1% vs 47.6%, p = 0.003)	No other parameter differed; no adverse events or trends in either group	Small; explicitly preliminary; no pre-specified non-inferiority margin; the authors state that more patients are needed to exclude a type II error	That a 3-day course was not shown to differ from 7 days in a small trial. It does not establish equivalence
Segel et al. (2003) [30]	Randomised clinical trial	PPROM; n = 48; GA range not stated in the published abstract	Ampicillin for 3 days (dose not stated in the published abstract)	Ampicillin for 7 days	Achievement of 7-day latency RR 0.83 (95% CI 0.51–1.38); chorioamnionitis and postpartum endometritis unchanged	Composite neonatal morbidity unchanged	Small; no pre-specified non-inferiority margin; the confidence interval includes clinically important harm	That shortening was not shown to change the rate of 7-day latency in a small trial. It does not establish equivalence
Sung et al. (2023) [32]	Single-centre RCT, per-protocol analysis	PPROM 22 0/7–33 6/7 wk; 151 randomised (75 vs 76), 124 analysed per protocol	IV cefazolin 1 g q12h + oral clarithromycin 500 mg q12h **until delivery**	Same regimen for 7 days	No difference in gestational age at delivery or rupture-to-delivery interval	RDS 32.8% vs 50.8% (p = 0.039) and composite neonatal morbidity 34.4% vs 53.9% (p = 0.026), both lower in the until-delivery arm; significance retained after multivariable adjustment; 12-month neurological outcomes unchanged	Single centre; per-protocol analysis; non-standard agents; mechanism unclear given unchanged latency	That continuing this regimen until delivery was associated with less neonatal morbidity than stopping at 7 days. It is one of two contemporary randomised duration comparisons and, despite important limitations, provides the more interpretable signal favouring continuation until delivery

Study (year)	Design	Population / GA / N	Regimen: agent, dose, route, duration	Comparator	Main obstetric outcomes	Main neonatal outcomes	Principal limitation	What the study actually establishes
Seaman et al. (2022) [15]	Systematic review and meta-analysis of 5 cohort studies	1289 women	Azithromycin-based latency regimens	Erythromycin-based regimens	Latency 6.7 vs 6.6 days, mean difference 0.07 days (95% CI −0.45 to 0.60); clinical chorioamnionitis pooled OR 0.53 (95% CI 0.39–0.71) favouring azithromycin	Not pooled	All included studies observational; no randomised data; no pre-specified non-inferiority margin	That observed latency is similar and chorioamnionitis may be lower with azithromycin. It does not demonstrate non-inferiority or superiority in a randomised sense
Pierson et al. (2014) [64]	Retrospective cohort	PPROM 24–34 wk; n = 168	Ampicillin + single oral azithromycin 1 g	Ampicillin + erythromycin	Latency 9.4 ± 10.0 vs 9.6 ± 13.2 days (p = 0.40)	No differences reported	Retrospective; powered only to detect a 5-day latency difference	Absence of a detected difference in an underpowered retrospective comparison
Appiagyei et al. (2017) [65]	Retrospective cohort, historical substitution	Singleton PPROM 23–33 6/7 wk; n = 162	β-lactam + single oral azithromycin 1 g	β-lactam + erythromycin	Median latency 5.86 (IQR 3.12–12.05) vs 6.37 (IQR 3.59–10.93) days (p = 0.75)	Secondary outcomes exploratory	Pre/post design; secular confounding	Absence of a detected latency difference after substitution
Martingano et al. (2020) [67]	Prospective observational cohort	PPROM ≥ 24 wk; n = 310	Azithromycin 1 g oral once + ampicillin/amoxicillin backbone	Erythromycin-containing regimen	Clinical chorioamnionitis 13.4% vs 25% (p = 0.010; adjusted RR 0.51, 95% CI 0.30–0.89); endometritis 14.8% vs 31% (adjusted RR 0.46, 95% CI 0.27–0.76); latency unchanged	Neonatal sepsis 4.9% vs 14.9% (adjusted RR 0.32, 95% CI 0.14–0.76)	Non-randomised; residual confounding	The largest observational signal identified favouring azithromycin on infectious outcomes. Hypothesis-generating, not confirmatory
Trahan et al. (2025) [13]	Multicentre retrospective cohort, 5 Canadian hospitals	PPROM 18–34 wk; n = 669	Macrolide alone (30%)	Ampicillin/amoxicillin + macrolide (50%) and other regimens	Macrolide alone: adjusted OR for latency ≤ 7 days 1.9 (95% CI 1.3–2.8); adjusted mean latency −6.7 days (95% CI −9.3 to −4.1); clinical chorioamnionitis 37% vs 14–25% (p = 0.001); histological chorioamnionitis 74% vs 52–61% (p < 0.001)	IVH 23% vs 11–13% (p = 0.009); ampicillin resistance in 11/19 (58%) neonatal bacteraemias	Retrospective; macrolide-alone use largely driven by reported penicillin allergy (confounding by indication); the ampicillin-resistance figure derives from 19 neonatal isolates in five Canadian centres over a single period and is not generalisable to other settings or eras	That macrolide monotherapy was associated with shorter latency and with higher rates of infectious and selected neonatal outcomes than β-lactam–macrolide combination therapy in contemporary practice; confounding by indication prevents causal inference. Ampicillin resistance among neonatal isolates was common in this cohort, but that estimate is centre- and era-specific and does not establish a resistance rate applicable to other PPROM populations
Kole-White et al. (2021) [28]	Retrospective historical-control cohort	PPROM < 34 wk; n = 116	Oral-only azithromycin + amoxicillin × 7 d	IV ampicillin + azithromycin × 2 d then oral × 5 d	Mean latency difference −0.15 days (95% CI −4.87 to 4.58)	Composite neonatal infection RR 0.43 (95% CI 0.05–3.52)	Very small; wide confidence intervals; historical controls	That an entirely oral regimen merits formal study. It does not establish equivalence of routes
Lee et al. (2016) [73]	Retrospective two-era cohort	PPROM < 34 wk; n = 314	Ceftriaxone 1 g IV q24h + clarithromycin 500 mg oral q12h + metronidazole 500 mg IV q8h, **until delivery**	Ampicillin ± cephalosporin ± macrolide/metronidazole (earlier era)	Median latency 23 (IQR 10–51) vs 12 (IQR 5–52) days, adjusted HR 0.69 (95% CI 0.49–0.96); delivery ≤ 7 d 20.2% vs 43.6%, adjusted OR 0.40 (95% CI 0.21–0.77); chorioamnionitis adjusted OR 0.30 (95% CI 0.16–0.57)	Funisitis adjusted OR 0.11 (95% CI 0.05–0.25); IVH 2.1% vs 19.0%, adjusted OR 0.08 (95% CI 0.02–0.35)	Two historical eras 1993–2003 vs 2003–2012; secular change in neonatal care is a major confounder; benefit concentrated in those with intra-amniotic infection/inflammation	That a broader-spectrum regimen continued until delivery is associated with longer latency in an era-comparison design. Not a randomised comparison
Day et al. (2024) [27]	Retrospective cohort	PPROM 22 0/7–33 6/7 wk; n = 296	Single-dose azithromycin + β-lactam	5-day azithromycin + β-lactam	Latency 5 vs 4 days (p = 0.641)	Histological chorioamnionitis significantly more frequent with the single dose (62.6% vs 46.4%, p = 0.006)	Retrospective; no pre-specified margin	That azithromycin dosing schedule may affect placental inflammation without affecting latency

Study (year)	Design	Population / GA / N	Regimen: agent, dose, route, duration	Comparator	Main obstetric outcomes	Main neonatal outcomes	Principal limitation	What the study actually establishes
Boelig et al., ADAPT trial (2024) [24]	Randomised phase I pharmacokinetic trial, halted early	PPROM; n = 6	Azithromycin 500 mg orally daily × 7 d	Azithromycin 1 g orally once	Not assessed	Not assessed	n = 6; pharmacokinetic endpoints only	That multi-day dosing sustains higher amniotic fluid concentrations. It establishes nothing about clinical outcome. The population pharmacokinetic model and the alternative dosing schedules simulated from this six-woman dataset [62] are correspondingly hypothesis-generating and do not constitute validated dose recommendations; macrolide activity is indexed to AUC/MIC, not to the time for which concentrations exceed a nominated threshold [74]
Sgayer et al. (2023) [70]	Three-centre randomised controlled trial	PPROM < 37 wk; n = 124	Cefuroxime + roxithromycin	Ampicillin + roxithromycin	Maternal infectious morbidity 6.5% vs 17.7% (one-sided p = 0.048); pathogen distribution in placental, membrane, cord and uterine cultures differed between arms	Not powered for neonatal outcomes	Small; one-sided significance testing at the conventional threshold; conducted by a single research group across three centres; not powered for neonatal outcomes	That a cefuroxime-based backbone reduces maternal infectious morbidity compared with ampicillin in a small randomised comparison. It is the only multicentre randomised comparison of β-lactam backbones identified in this review, and it does not establish neonatal benefit
Shabana Hameed et al. (2025) [33]	Single-centre randomised controlled study	PPROM 26 0/7–36 6/7 wk; n = 110 (55 vs 55)	Fixed course: IV ceftriaxone + metronidazole, then oral clarithromycin to complete 7 days	Continuation: same regimen with oral clarithromycin continued until delivery	No maternal complications in either arm; persistent amniotic fluid leakage 56.4% with the fixed 7-day course vs 81.8% with continuation until delivery (p < 0.002)	NICU admission 47.3% (fixed 7-day) vs 41.8% (continuation), p = 0.56; neonatal stay of 1–3 days in 52.7% vs 30.9% (p = 0.02); four neonatal deaths, all in infants below 1000 g, in the fixed 7-day arm and none with continuation	Marked baseline imbalance: cervix closed at admission in 76.3% of the fixed 7-day arm vs 20.0% of the continuation arm, which raises concern about the effectiveness of the randomisation and allocation procedures; allocation described only as simple random sampling; the abstract and the concluding section draw opposite inferences from the same results	That the study did not demonstrate a clear clinical advantage of continuation until delivery over a fixed 7-day course. The trial was not designed as a non-inferiority study and pre-specified no margin. Marked baseline imbalance and other methodological limitations prevent a reliable causal interpretation, and the reported direction of effect is opposite to that of the Korean trial
Frank Wolf et al. (2020) [69]	Single-centre randomised trial	PPROM; n = 84	Cefuroxime + roxithromycin	Ampicillin + roxithromycin	Median latency 4.63 vs 2.30 days (p = 0.039)	Not reported separately	Small; single centre; open allocation not described in detail; not powered for neonatal outcomes	That a cefuroxime-based backbone prolonged latency relative to ampicillin in a small randomised comparison. It does not establish neonatal benefit
Musavi et al. (2022) [17]	Open-label randomised clinical trial	Prelabour rupture of membranes, mixed preterm and near-term; n = 194; single centre (Tehran, Iran)	Oral azithromycin 1 g + IV ampicillin 2 g q6h × 48 h, then oral amoxicillin 500 mg q8h × 5 d	IV erythromycin 400 mg q6h × 7 d + IV ampicillin 2 g q6h × 48 h, then oral amoxicillin 500 mg q6h × 5 d	Latency interval was the stated primary outcome; the point estimates could not be verified because the full text was not retrievable	Neonatal outcomes stated as secondary; not verifiable from the accessible record	Open-label; single centre; population not restricted to PPROM before 34 weeks; no pre-specified non-inferiority margin; not indexed in MEDLINE; full text not retrievable for independent verification	That the azithromycin-for-erythromycin substitution has been examined in a randomised design. It does not establish non-inferiority, and its numerical results could not be independently verified
Karataş Gezen and Karaaslan (2026) [59]	Single-centre randomised controlled trial	PPROM 34–37 wk; n = 40 (20 vs 20)	Ampicillin–sulbactam + azithromycin + amoxicillin	Ceftriaxone + clarithromycin + amoxicillin	Clinical chorioamnionitis 20.0% vs 5.0% (p = 0.141); no significant difference in inflammatory markers or mode of delivery	Neonatal intensive care stay 8.3 ± 6.7 vs 4.3 ± 1.9 days (p = 0.356)	n = 40; no sample-size calculation; registered after recruitment began (NCT07148622); late-preterm population in which several societies do not recommend latency antibiotics	That two multi-agent regimens were compared in a randomised design at 34–37 weeks without a detected difference. The trial is too small for any inference of equivalence and has limited external validity for PPROM before 34 weeks

Abbreviations: AUC, area under the concentration–time curve; CI, confidence interval; GA, gestational age; GBS, group B *Streptococcus*; HR, hazard ratio; IQR, interquartile range; IV, intravenous; IVH, intraventricular haemorrhage; MIC, minimum inhibitory concentration; NEC, necrotising enterocolitis; NNH, number needed to harm; OR, odds ratio; PPROM, preterm prelabour rupture of membranes; qds, four times daily; q6h/q8h/q12h/q24h, every 6/8/12/24 hours; RCT, randomised controlled trial; RDS, respiratory distress syndrome; RR, risk ratio; wk, weeks.

Table S2. Contemporary international guideline recommendations for latency antibiotic therapy in preterm prelabour rupture of membranes: gestational-age range, agent, dose, route, duration, alternatives, allergy and previable guidance, evidence basis, and residual uncertainty.

Organisation (document, year)	Gestational-age range	First-line agent and dose	Route	Duration	Alternative regimen	Allergy considerations	Previable/periviable PPROM	Main evidence basis	Residual uncertainty
ACOG, Practice Bulletin No. 217 (2020) [1]	< 34 0/7 wk (Level A); latency antibiotics not appropriate at 34 0/7–36 6/7 wk	Ampicillin 2 g q6h + erythromycin 250 mg q6h, then amoxicillin 250 mg q8h + erythromycin base 333 mg q8h	IV × 48 h, then oral	7 days total	Azithromycin (e.g. single oral 1 g dose) where erythromycin is unavailable or not tolerated	"Another agent effective against GBS" may replace the β-lactam; no specific agent or dose named	Broad-spectrum antibiotics reasonable to consider if expectant management is chosen	NICHD trial; ORACLE I	No comparative evidence for the substitution it permits; no allergy regimen specified
SMFM, Consult Series #71 (2024) [34]	≥ 24 0/7 wk: recommended (GRADE 1B). 20 0/7–23 6/7 wk: may be considered (GRADE 2C)	As ACOG (IV ampicillin + erythromycin × 48 h, then oral amoxicillin + erythromycin × 5 d)	IV then oral	7 days	Azithromycin where erythromycin unavailable	Not specified for true β-lactam allergy	Explicitly addressed; caution against prolonged or repeated courses on stewardship grounds	Observational data for the previable window; NICHD trial above 24 wk	States that optimal antibiotic type, dose and timing are unknown in this population
RCOG, Green-top Guideline No. 73 (2019) [86]	From 24+0 wk (guideline scope)	An antibiotic, preferably erythromycin (dose not stated in the summary recommendation)	Oral	10 days or until established labour, whichever is sooner	Not named in the summary recommendation	Not addressed in the summary recommendation	Out of scope	ORACLE I	No β-lactam–macrolide combination option; allergy gap. Guideline reviewed in October 2024 and extended for two years, with the next review commencing in 2026
NICE, NG25 (2015, recommendations amended 2019 and 2022) [87]	PPROM, termed P-PROM by NICE (guideline scope)	Erythromycin 250 mg four times daily (1.4.1)	Oral	Maximum 10 days or until established labour	Oral penicillin if erythromycin is contraindicated or not tolerated (1.4.2)	As above	Not separately specified	ORACLE I	Recommendation content unchanged since 2015 apart from licensing updates
WHO, Recommendations on interventions to improve preterm birth outcomes (2015) [88]	PPROM; antibiotics not recommended for near-term PPROM with immediate induction	Erythromycin 250 mg four times daily (conditional recommendation, moderate-quality evidence)	Oral	10 days or until delivery	Not specified	Not specified	Not addressed	ORACLE I and pooled evidence	Conditional grading; no dose comparison; no update to the antibiotic recommendation identified since 2015
FIGO, Good practice recommendations (2023) [100]	Confirmed PPROM; no gestational-age restriction stated for the antibiotic recommendation	Erythromycin 250 mg every 6 hours	Route not stated in the recommendation box	Up to 10 days or until established labour	If erythromycin is contraindicated or the patient is allergic to it: intravenous ampicillin 2 g every 6 hours followed by oral amoxicillin 250 mg every 8 hours for 5 days — that is, a β-lactam without a macrolide	Addresses erythromycin allergy only; β-lactam allergy not addressed	Not separately addressed for antibiotics	ORACLE I; NICHD trial	Co-amoxiclav explicitly to be avoided because of necrotising enterocolitis. No formal evidence grading is applied to the recommendation

Organisation (document, year)	Gestational-age range	First-line agent and dose	Route	Duration	Alternative regimen	Allergy considerations	Previable/periviable PPROM	Main evidence basis	Residual uncertainty
CNGOF (2018) [31]	From the time PPROM is diagnosed; the same protocol applies before viability	Amoxicillin, a third-generation cephalosporin or erythromycin as monotherapy, or erythromycin combined with amoxicillin (professional consensus)	Amoxicillin and erythromycin parenteral or oral; third-generation cephalosporin parenteral	7 days (Grade C); early discontinuation is acceptable if the initial vaginal culture is negative (professional consensus)	The erythromycin–amoxicillin combination is the listed alternative to monotherapy	Not addressed for prophylaxis; aztreonam is named only for severe β-lactam allergy when treating established intra-uterine infection	Same protocol as at later gestations (professional consensus)	National synthesis of the same trials; prophylaxis itself Grade A	No doses are specified anywhere in the guideline. The only guideline in this table that permits third-generation cephalosporin monotherapy and stopping early on a microbiological criterion. Explicitly advises against co-amoxiclav, aminoglycosides, glycopeptides, first- and second-generation cephalosporins, clindamycin and metronidazole
RANZCOG, Best Practice Statement (2021, review due 2024) [110]	Up to 36+6 wk	Option 1: erythromycin 250 mg four times daily. Option 2: amoxicillin/ampicillin 2 g IV q6h × 48 h then amoxicillin 250 mg q8h × 5 d **plus** erythromycin 250 mg q6h × 10 d	Oral, or IV then oral	10 days (option 1); 7-day β-lactam with 10-day erythromycin (option 2)	Azithromycin may be considered in place of a multi-day erythromycin course	Defers to national therapeutic guidelines for penicillin hypersensitivity	Not specified	Both landmark trials	Two structurally different regimens endorsed side by side; document past its own review date
DGGG/OEGGG/SGGG, S2k guideline, AWMF 015/025, September 2022 [109]	22+0–33+6 wk	IV ampicillin for 2 days, then oral amoxicillin for 5 days, plus oral azithromycin; amoxicillin must not be combined with clavulanic acid	IV then oral	7 days for the β-lactam component	—	Not specified	Antibiotic treatment may be considered before viability	Expert consensus	States explicitly that no clear recommendation can be made for the dose or duration of azithromycin; the only guideline in this table advising the same antibiotic management at 34+0–36+6 wk
SOGC, Guideline No. 430 (November 2022) [111]	All patients with PPROM < 37 wk	Either a macrolide alone (erythromycin, azithromycin or clarithromycin), with group B streptococcal cover for 2 days where GBS status is positive or unknown, or a combination of ampicillin/amoxicillin with a macrolide irrespective of GBS status; no doses are specified (strong, moderate)	Not specified in the recommendation	No data support treatment beyond 10 days (strong, moderate)	Alternative antibiotic therapy may be considered on the basis of local resistance data (conditional, low)	Not separately addressed; the requirement is that the regimen cover GBS where status is positive or unknown	Previable PPROM is addressed in the guideline as a whole; no separate antibiotic recommendation for that window	GRADE-graded synthesis of randomised trials, meta-analyses and observational studies searched to 2021	States that the optimal antibiotic regimen remains unclear. Specifies no doses. SOGC policy is to review content 5 years after publication, so the document is due for review in 2027

Abbreviations: GBS, group B *Streptococcus*; GRADE, Grading of Recommendations Assessment, Development and Evaluation; IV, intravenous; NICHD, Eunice Kennedy Shriver National Institute of Child Health and Human Development; PPROM, preterm prelabour rupture of membranes; q6h/q8h, every 6/8 hours; wk, weeks. Evidence grades reflect each society's confidence in offering latency antibiotics, not head-to-head confirmation that the graded regimen is superior to alternatives. A systematic review of national and international PPROM guidelines has been published separately [9]; this table complements it with dose, route, allergy and previable/periviable columns and with an explicit statement of residual uncertainty for each document.
